# Neutrophil Extracellular Traps in Exocrine Pancreatic Disease: A Comprehensive Review of Pathogenesis, Severity Stratification, and Therapeutic Targeting

**DOI:** 10.3390/cells15050440

**Published:** 2026-02-28

**Authors:** Vesna Vulovic, Bojan Stojanovic, Ivan Jovanovic, Milica Dimitrijevic Stojanovic, Bojana S. Stojanovic, Jasna Gacic, Simona Petricevic, Jelena Kostic, Novica Nikolic, Snezana Lukic, Slobodan Todorovic, Ana Sekulic, Milena Vuletic, Miladin Boskovic, Tatjana Lazarevic

**Affiliations:** 1 Department of Surgery, University Hospital Medical Center Bežanijska Kosa, 11000 Belgrade, Serbia; 2 Department of Surgery, Faculty of Medical Sciences, University of Kragujevac, 34000 Kragujevac, Serbia; bojan.stojanovic01@gmail.com (B.S.);; 3 Center for Molecular Medicine and Stem Cell Research, Faculty of Medical Sciences, University of Kragujevac, 34000 Kragujevac, Serbia; 4 Department of Pathology, Faculty of Medical Sciences, University of Kragujevac, 34000 Kragujevac, Serbia; 5 Department of Pathophysiology, Faculty of Medical Sciences, University of Kragujevac, 34000 Kragujevac, Serbia; 6 Department of Surgery with Anesthesiology, Faculty of Medicine, University of Belgrade, 11000 Belgrade, Serbia; 7 Department of Anesthesiology and Intensive Care, University Hospital Medical Center Bežanijska Kosa, 11000 Belgrade, Serbia; 8 Department of Radiology, Faculty of Medical Sciences, University of Kragujevac, 34000 Kragujevac, Serbia; 9 Department of Internal Medicine, Faculty of Medical Sciences, University of Kragujevac, 34000 Kragujevac, Serbia

**Keywords:** neutrophil extracellular traps, NETosis, acute pancreatitis, severe acute pancreatitis, pancreatic ductal adenocarcinoma, immunothrombosis, desmoplasia, metastasis, biomarkers, therapeutic targeting

## Abstract

**Highlights:**

**What are the main findings?**

- NETs act as a shared mechanistic thread across exocrine pancreatic diseases, linking sterile inflammation with thromboinflammation and tissue remodeling in both acute pancreatitis and pancreatic cancer.
- In acute pancreatitis, early and excessive NET formation aligns with more severe clinical trajectories and complications, including microvascular thrombosis, ductal obstruction, and systemic organ dysfunction.

**What are the implications of the main findings?**

- NET-related circulating and tissue biomarkers (e.g., cfDNA-, MPO–DNA-, and CitH3-based readouts) may support earlier risk stratification and monitoring of therapeutic response in pancreatic disease.
- Therapeutically targeting NET biology (e.g., NET dismantling with DNase or upstream pathway inhibition such as PAD4/autophagy/redox signaling) is a rational strategy to reduce thrombosis and tissue injury and may help rebalance antitumor immunity in PDAC.

**Abstract:**

Neutrophil extracellular traps (NETs) are web-like DNA–protein structures released by activated neutrophils. Initially recognized for their antimicrobial roles, NETs are now known to drive sterile inflammation, thrombosis, and tissue remodeling. This review highlights their involvement in key pancreatic diseases, including acute pancreatitis (AP) and pancreatic ductal adenocarcinoma (PDAC). In AP, early NET formation correlates with disease severity and septic complications, contributing to acinar injury, microvascular thrombosis, ductal obstruction, and organ dysfunction. In PDAC, NETs shape a fibrotic and immune-resistant tumor microenvironment by promoting stromal activation, immune exclusion, metastasis, and hypercoagulability. Tumor- and stroma-derived signals sustain NET formation within this niche. We also discuss NET-related biomarkers for risk assessment and therapy monitoring, and explore therapeutic strategies that target NETs—ranging from their degradation with DNase to their inhibition of upstream pathways such as PAD4, autophagy, and oxidative signaling. Targeting NETs may also reduce their downstream effects on thrombosis and immune suppression. Overall, NETs emerge as critical drivers of pancreatic disease progression and represent promising therapeutic targets.

## 1. Introduction

Neutrophil extracellular trap formation has moved from a host-defense concept to a unifying pathobiological mechanism that links sterile pancreatic inflammation, tissue remodeling, and cancer-associated immune dysregulation. NETs are DNA–histone scaffolds decorated with neutrophil enzymes and alarmins, generated through regulated programs collectively termed NETosis, and their persistence can amplify inflammation and immunothrombosis beyond antimicrobial benefit. In pancreatic diseases, this balance is particularly consequential: acute pancreatitis is initiated by inappropriate intrapancreatic zymogen activation, yet clinical trajectory is largely shaped by the ensuing innate immune response, in which neutrophils and NET products can intensify local injury and propagate systemic inflammatory complications. This review therefore focuses on NETosis as a mechanistic thread across pancreatic disorders, integrating how NET components and upstream triggers shape acinar and ductal pathology in pancreatitis, and how NET-rich inflammatory circuits remodel the pancreatic tumor microenvironment to favor immune exclusion, stromal activation, thrombosis, and metastatic competence in pancreatic malignancies. We first summarize core NET biology and key regulatory pathways, then examine disease-specific evidence in pancreatitis phenotypes (including necrotizing and metabolic forms), and finally synthesize NET-driven mechanisms in PDAC and related pancreatic tumors with an emphasis on translational opportunities for biomarker development and therapeutic interception.

## 2. Neutrophils and NETosis: Core Concepts and Mechanistic Framework

Neutrophils are central effectors of innate immunity and are rapidly mobilized to inflamed tissues, where they provide an early line of host defense [1]. In the circulation, they constitute the largest leukocyte fraction, accounting for roughly 50–70% of total white blood cells, and they are characterized by a short lifespan that is typically under 24 h [2]. Under physiological conditions, neutrophils help contain acute inflammatory responses and protect against extracellular pathogens through direct microbicidal activity and through coordinated communication with other immune compartments, including the release of chemotactic and immunomodulatory mediators that amplify and shape downstream cellular recruitment and activation [2].

Neutrophils execute their effector functions through several tightly coordinated programs, including pathogen and debris clearance by phagocytosis, generation of reactive oxygen species (ROS), regulated release of proteases and other cytotoxic mediators from granules, and paracrine signaling that activates and recruits additional immune cell populations [3,4]. In oncology, tumor-associated neutrophils (TANs) can display context-dependent behavior: under certain conditions they support anti-tumor immunity, whereas in others they facilitate tumor progression by sustaining inflammation, remodeling extracellular matrices, promoting angiogenesis, and enabling invasion and dissemination [5,6,7]. In tissue repair, neutrophils are similarly indispensable early responders, where they remove contaminants and necrotic material and provide molecular cues that coordinate subsequent phases of wound healing and regeneration [8,9].

Neutrophils deploy three principal antimicrobial strategies: phagocytosis, degranulation, and the release of NETs, web-like extracellular structures extruded by activated neutrophils [1]. NETs are composed of decondensed chromatin that is coated with proteins originating from neutrophil granules and cytosol. Many of these proteins maintain their antimicrobial properties, allowing NETs to trap and neutralize pathogens in the extracellular space [10]. Beyond infection biology, NETs are increasingly recognized as relevant to cancer because they can modify tissue inflammatory cues and influence tumor–host interactions within the tumor microenvironment [11].

### 2.1. Neutrophil Extracellular Traps: Biology, Mechanisms of Formation, and Pathophysiologic Relevance

Neutrophil extracellular traps were first described in 2004, when Brinkmann and colleagues reported that neutrophils exposed to acute bacterial infection can release extracellular, net-like structures composed of chromatin fibers decorated with antimicrobial proteases, including neutrophil elastase [12]. Follow-up work expanded this initial observation by showing that NET release is not restricted to bacterial triggers, but can also be elicited during infections caused by fungi, viruses, and parasites [13,14,15,16,17]. Functional studies subsequently supported the concept that NETs contribute to host defense by restraining pathogens and limiting their dissemination, and by participating in coordinated immunothrombotic responses in severe infection, including sepsis, where NETs interact with platelet innate-sensing pathways such as Toll-like receptor 4 (TLR-4) [12,18].

NETs are extracellular, web-like scaffolds released by activated neutrophils that consist primarily of decondensed DNA and histones, interlaced with antimicrobial components derived from neutrophil granules and cytosol [1,12,19]. This composite matrix functions as an adhesive platform that immobilizes microbes and concentrates toxic effector molecules in their immediate vicinity [1]. Beyond nucleic acids and core histones (H2A, H2B, H3, and H4), NETs commonly carry canonical granule enzymes such as neutrophil elastase (NE), myeloperoxidase (MPO), and proteinase 3 (PR3), as well as additional alarmins and structural proteins that may be co-released depending on the stimulus [1,20]. Importantly, NE and MPO are not only abundant NET constituents but also participate in the intracellular events that enable NET release, linking granule biology with extracellular trap deployment [21].

Proteomic profiling has highlighted that NET composition is broader than a small set of signature proteins and varies with the activating signal. Early analyses of phorbol 12-myristate 13-acetate (PMA)-induced NETs identified a limited core group of proteins, but subsequent high-depth studies expanded the NET proteome substantially, reporting hundreds of associated proteins with a recurrent subset detected across preparations [22]. In addition to conserved nuclear and granule components, NETs may incorporate cytosolic antimicrobial factors such as calprotectin and lactoferrin, and several other bactericidal proteins have been reported on NET strands, including azurocidin, cathelicidin, lysozyme, and bactericidal/permeability-increasing proteins (BPI family) [1,13]. These observations support a model in which NETs represent a structured extracellular compartment that concentrates diverse neutrophil-derived antimicrobial and immunomodulatory molecules, with composition shaped by context and stimulus rather than being invariant.

NETosis denotes the cellular program that culminates in the generation of NETs [23]. It was initially viewed as a distinct neutrophil death pathway, separate from apoptosis and necrosis, and is now framed more precisely as a regulated response in which NET release may, but does not necessarily, end in cell death [10,11]. In its strictest sense, NETosis refers to NET formation accompanied by neutrophil death, and it is commonly driven by intracellular oxidant signaling, particularly ROS [24,25]. A broad spectrum of triggers can initiate this program, including microbial products, immune complexes, activated platelets, and experimental agonists such PMA or lipopolysaccharide (LPS) [11,26]. Across these stimuli, a central mechanistic requirement is nuclear chromatin decondensation, which converts compacted lobulated neutrophil chromatin into an expandable scaffold that can be externalized as NETs [27].

**Figure 1:**
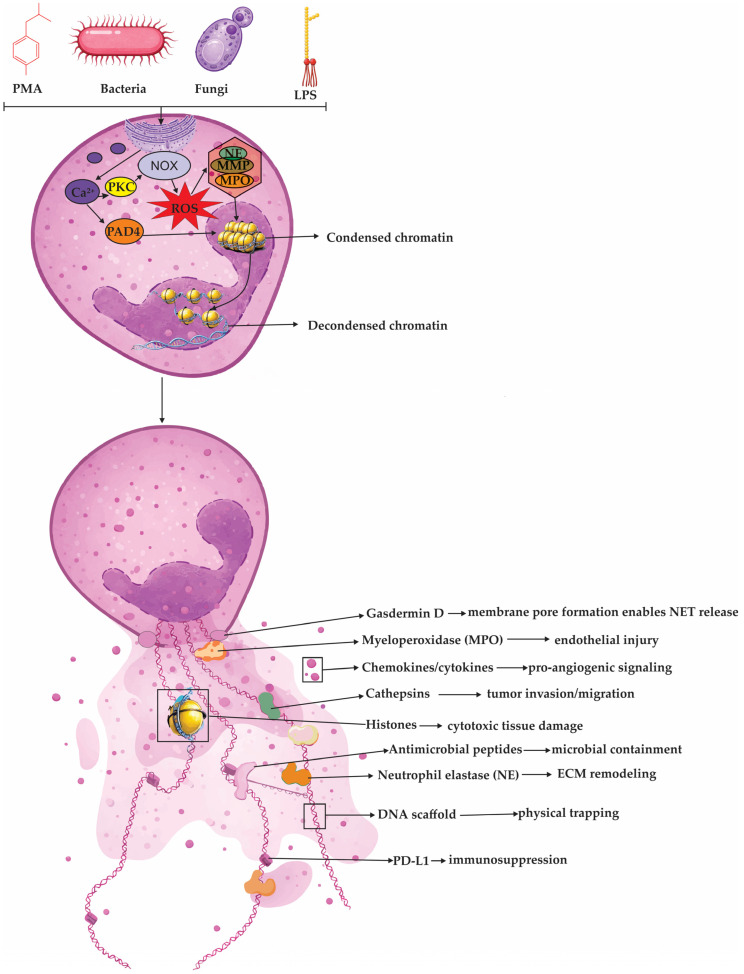
Key triggers, intracellular execution pathways, and functional cargo of neutrophil extracellular traps (NETs). Diverse stimuli, including experimental agonists (PMA), microbial pathogens (bacteria and fungi), and pathogen-associated molecular patterns (LPS), can initiate NET formation. In the canonical pathway, Ca^2+^-dependent signaling and protein kinase C (PKC) activation promote NADPH oxidase (NOX2)-driven reactive oxygen species (ROS) generation, facilitating mobilization of granular enzymes (e.g., neutrophil elastase, myeloperoxidase) and PAD4-mediated histone citrullination, which together drive chromatin decondensation. Subsequent membrane permeabilization, including gasdermin D-associated pore formation, enables extracellular release of decondensed chromatin decorated with antimicrobial and immunomodulatory proteins. The released NET scaffold can mediate pathogen trapping and containment while also exerting context-dependent tissue and tumor-related effects through cargo components such as histones, proteases (e.g., neutrophil elastase, cathepsins), MPO, chemokines/cytokines, and immune-regulatory signals (e.g., PD-L1), thereby linking innate defense with inflammation, vascular injury, extracellular matrix remodeling, angiogenic signaling, and immunosuppression.

At the molecular level, chromatin decondensation during NETosis is driven by a central enzymatic triad: peptidylarginine deiminase 4 (PAD4), NE, and MPO [28]. Upon NET-inducing stimulation, a rapid surge in intracellular calcium (Ca^2+^) activates the calcium-dependent enzyme PAD4 [29]. This enzyme catalyzes histone citrullination, most notably detectable as citrullinated histone H3 (Cit-H3), by converting arginine residues into citrulline [29,30]. This biochemical modification neutralizes the positive charge of arginine, weakening the electrostatic interactions that normally anchor DNA to histones. As a result, the chromatin begins to unravel and swell, a critical structural change that enables the mixing of decondensed chromatin with cytoplasmic and granular proteins, ultimately facilitating the release of NETs [30]. In addition to Ca^2+^-activated PAD4-mediated histone citrullination, NETosis-associated chromatin decondensation is often amplified by ROS-dependent mobilization and nuclear translocation of azurophilic granule enzymes [11]. Namely, in the canonical NADPH oxidase (NOX2)-dependent pathway, neutrophil activation drives assembly of NOX2 and an early burst of ROS, which functions as a signaling cue that releases azurophilic granule enzymes from their compartments and enables their redistribution toward the nucleus [11]. Under these oxidative conditions, NE translocates into the nucleus and proteolytically processes histones, loosening nucleosomal packing and initiating chromatin decondensation [31]. Myeloperoxidase follows and cooperates with NE to amplify chromatin relaxation, in part by promoting steps that further destabilize nucleosomes and facilitate chromatin remodeling [31,32]. As histone–DNA constraints progressively weaken, the chromatin swells and nuclear architecture becomes increasingly permissive for NET formation and release [28]. An overview of major NET-inducing stimuli and the canonical Ca^2+^/PKC–NOX2-ROS–PAD4 pathway culminating in chromatin decondensation and release of NET-associated effector cargo is summarized in Figure 1.

**Figure 2:**
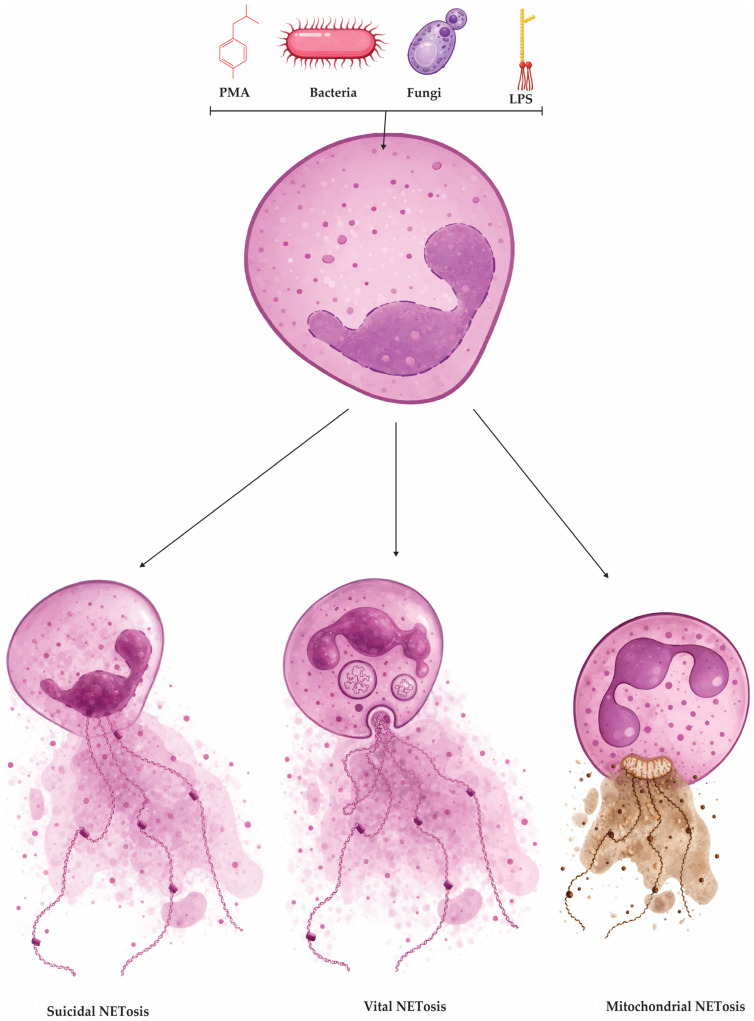
Operational forms of NETosis: suicidal, vital, and mitochondrial NET release. NET formation can proceed through distinct stimulus- and context-dependent programs. Suicidal (lytic) NETosis culminates in progressive chromatin decondensation followed by nuclear and plasma membrane rupture, releasing nuclear DNA–histone scaffolds and granular proteins into the extracellular space and resulting in neutrophil death. Vital (non-lytic) NET release describes rapid extrusion of NET material while preserving key neutrophil functions, including motility and phagocytosis, thereby allowing continued cellular activity after NET deployment. Mitochondrial NET release involves extracellular expulsion of mitochondrial DNA that contributes to the NET scaffold, typically in settings linked to calcium-dependent signaling and mitochondrial oxidant stress and may occur with reduced reliance on classical NOX2-derived ROS. Collectively, these pathways highlight that NET generation is not a single linear cascade but a flexible effector response shaped by the initiating stimulus and the inflammatory microenvironment.

Alternative routes also exist, including pathways that rely more on mitochondrial ROS or that proceed with minimal detectable ROS in specific contexts, underscoring that NET formation is stimulus- and context-dependent rather than a single linear cascade [25]. Consistent with this biology, NETosis is often categorized into three operational forms: suicidal (lytic) NETosis, in which nuclear and plasma membrane rupture leads to NET release and neutrophil death; vital (non-lytic) NET release, characterized by rapid extrusion of NET material while preserving key neutrophil functions such as motility and phagocytosis; and mitochondrial NET release, where mitochondrial DNA contributes to the extracellular scaffold in settings frequently linked to calcium signaling and mitochondrial oxidant stress [11]. The major operational NETosis phenotypes—suicidal (lytic), vital (non-lytic), and mitochondrial NET release—are schematically summarized in Figure 2.

NET clearance remains incompletely defined, but available data indicate that NETs can persist for days in inflamed tissues and are progressively dismantled by endogenous nucleases, particularly deoxyribonuclease I (DNase I) [33]. Experimental DNase I administration rapidly fragments the DNA backbone of NETs, yet protein constituents may remain after DNA degradation, implying that complete resolution requires additional disposal pathways [33]. In this context, extracellular DNase-driven fragmentation likely “pre-processes” NETs into smaller, phagocytosis-competent fragments, and DNase I has been shown to increase macrophage uptake of NET material in vitro, supporting a cooperative model in which nuclease activity and efferocytosis-like phagocytosis jointly clear NET remnants and terminate their inflammatory signaling [33,34]. Because NETs are DNA–protein complexes, their detection in biofluids and tissues typically relies on surrogate biomarkers, including cell-free DNA (cfDNA) and NET-associated proteins such as myeloperoxidase MPO, neutrophil NE, and citrullinated histone H3 (citH3), as well as more specific composite readouts that capture DNA–protein co-localization (e.g., MPO–DNA or citH3–DNA complexes) measured by immunoassays [35]. In plasma, serum, or neutrophil culture supernatants, cfDNA and MPO–DNA are commonly used to estimate NET burden, whereas immunohistochemistry (IHC) and immunofluorescence (IF) in tissue sections can visualize MPO, NE, and citH3 with spatial context [35]. Notably, no single method has been universally accepted as a definitive standard; thus, combining orthogonal markers and, when feasible, pairing biochemical assays with imaging-based confirmation is generally considered the most robust strategy for NET assessment.

#### 2.1.1. Suicidal (Lytic) NETosis

Suicidal (lytic) NETosis is a time-dependent, regulated neutrophil death program that typically unfolds over 1–4 h after stimulation and results in NET release coupled to cell lysis [11,36]. It can be initiated by diverse cues, including microbes (bacteria, fungi, viruses), immune complexes and autoantibodies, inflammatory mediators such as tumor necrosis factor alpha (TNF-α), and oxidant stress (e.g., hydrogen peroxide) [13,14,37]. A frequently used experimental trigger is PMA, which activates protein kinase C (PKC) and downstream mitogen-activated protein kinase (MAPK) signaling (Raf–MEK–ERK: rapidly accelerated fibrosarcoma kinase–MAPK/ERK kinase–extracellular signal-regulated kinase), promoting assembly of the NADPH oxidase 2 (NOX2) complex (gp91phox/NOX2 with p22phox, p47phox, p40phox, p67phox) and a robust ROS burst [31,38,39]. ROS signaling, together with calcium influx PAD4, leading to histone citrullination and weakening histone–DNA binding, a key prerequisite for chromatin relaxation [25]. In parallel, azurophilic granules mobilize NE and MPO. Neutrophil elastase translocates to the nucleus to proteolyze histones, while MPO amplifies chromatin remodeling, collectively driving profound chromatin decondensation [27,32]. As nuclear architecture collapses, nuclear envelope integrity is lost and chromatin mixes with granule-derived and cytosolic proteins to form mature extracellular traps [33]. Terminal NET release represents a final “barrier” phase in which decondensed chromatin is ready to exit, but remains physically confined first by the nuclear envelope and then by the plasma membrane [31]. In lytic NETosis, nuclear rupture is facilitated when the nuclear lamina is destabilized through phosphorylation-driven disassembly of lamins, which weakens the envelope’s mechanical support and makes it prone to tearing; here, upstream NET-inducing signals that elevate intracellular Ca^2+^ and activate kinase cascades can engage protein kinase Cα (PKCα), promoting its nuclear translocation and phosphorylation of lamin B, a modification that triggers lamina disassembly and nuclear envelope breakdown [27,31]. Cell-cycle-associated kinases, including cyclin-dependent kinases 4 and 6 (CDK4/6), have been implicated as additional lamin-targeting activities that further lower the threshold for nuclear rupture and accelerate chromatin access to the cytoplasm [40]. Once the nuclear barrier fails, chromatin spills into the cytoplasm, where it can mix with granular and cytosolic proteins (including NE and MPO) to form the DNA–protein scaffold that will become the NET [11]. The remaining obstacle is the plasma membrane, and loss of membrane integrity can be promoted by pore-forming and lytic programs: gasdermin D (GSDMD) can become a membrane pore-forming effector after proteolytic cleavage, which in neutrophils may occur in a stimulus-dependent manner and can involve NE, thereby increasing membrane permeability and facilitating extrusion [41,42]. Concomitant breakdown of the cortical cytoskeleton reduces the mechanical resistance of the plasma membrane, allowing the swollen chromatin mass to breach the cell boundary, culminating in extracellular release of DNA decorated with antimicrobial enzymes and irreversible neutrophil death [43].

#### 2.1.2. Vital (Non-Lytic) NET Release

Vital (non-lytic) NET release refers to a rapid form of NET formation in which neutrophils externalize NET material while preserving plasma membrane integrity and remaining functionally active [28,44]. In this program, NETs are often exported through nuclear envelope remodeling with vesiculation and vesicular trafficking rather than through catastrophic membrane rupture, and it can proceed with minimal or no dependence on NOX2-derived reactive oxygen species [11,45]. Reported triggers include certain bacteria and bacterial products, activated platelets, and complement-related signals; in sepsis models, platelet activation through TLR-4 and platelet–neutrophil interactions have been linked to NET release occurring within minutes [1,46]. Similarly, during skin infection with Gram-positive organisms such as *Staphylococcus aureus*, extracellular trap release has been observed within 5–60 min and may involve pattern-recognition receptor signaling (e.g., TLR-2) rather than an overt oxidative burst [47]. A common mechanistic feature across several NOX-independent contexts is a rise in intracellular calcium, which activates PAD4 and promotes histone citrullination, weakening histone–DNA interactions and enabling chromatin expansion [11]. The extruded chromatin then acquires granule- and cytosol-derived proteins before being released by exocytic pathways, allowing neutrophils to remain viable and to retain key effector behaviors such as chemotaxis and phagocytosis after NET deployment [48].

#### 2.1.3. Mitochondrial NET Release

A third NET-generating program produces extracellular traps in which the DNA backbone is predominantly mitochondrial DNA (mtDNA) rather than nuclear chromatin, and it can be rapidly elicited by inflammatory cues such as complement component 5a (C5a) and, in some settings, LPS, with mitochondrial ROS (mtROS) acting as the dominant execution signal [49,50]. Unlike NOX2-driven lytic NETosis, this “vital” mtDNA-NET response typically preserves overall plasma membrane integrity because it bypasses the full sequence of nuclear envelope breakdown and terminal rupture, instead mobilizing mtDNA from mitochondria for export [49]. The extrusion process is energetically demanding and depends on glycolysis-derived adenosine triphosphate (ATP), which fuels coordinated cytoskeletal remodeling: reorganization of microtubules together with filamentous actin (F-actin) dynamics, that provides the trafficking routes and mechanical force needed to externalize DNA-containing material while keeping the cell viable [51]. The same cytoskeletal program also promotes degranulation and spatially couples released granule proteins to the outgoing mtDNA, generating an antimicrobial DNA–protein mesh [43]. Once outside the cell, mtDNA is not merely structural: because it is cytosine–phosphate–guanine (CpG)-rich and often oxidatively modified under stress, it behaves as a danger-associated molecular pattern (DAMP), and when mtDNA is internalized by responder cells and gains access to the cytosol it can bind cyclic guanosine monophosphate–adenosine monophosphate synthase (cGAS), induce 2′3′-cyclic guanosine monophosphate–adenosine monophosphate (2′3′-cGAMP), activate stimulator of interferon genes (STING) on the endoplasmic reticulum, and drive TANK-binding kinase 1–interferon regulatory factor 3 (TBK1–IRF3) signaling with downstream type I interferon and inflammatory transcriptional outputs, a mechanism frequently invoked to explain immune amplification in disorders such as systemic lupus erythematosus (SLE) [52,53,54]. Mechanistically, rapid mtNET induction has been linked to Ca^2+^-sensitive ion channel activity that couples extracellular triggers to mitochondrial stress responses, thereby promoting mtROS generation and making mtDNA accessible for release, while emerging checkpoint regulators refine this output in specific contexts: Sirtuin 1 (SIRT1) has been proposed to gate mtDNA release via mitochondrial permeability transition pore (mPTP) opening in tumor-associated aged neutrophils, and optic atrophy 1 (OPA1) supports mitochondrial fitness and ATP supply that sustain the microtubule- and actin-dependent export machinery required for mtDNA extrusion [55,56].

#### 2.1.4. Autophagy as a Regulatory Layer in NET Formation

Beyond the canonical NETosis programs, autophagy is increasingly viewed as a modulatory layer that adjusts how readily neutrophils form NETs by integrating metabolic status with redox and trafficking demands [57,58]. In many experimental settings, intact autophagic flux appears permissive for NET release, whereas pharmacologic inhibition or genetic disruption of autophagy-related machinery reduces NET formation, consistent with the concept that autophagy helps sustain the bioenergetic, organelle-traffic, and cytoskeletal requirements of this response [57]. Mechanistically, this relationship is often organized around the phosphoinositide 3-kinase (PI3K)–Akt–mechanistic target of rapamycin (mTOR) axis, a central nutrient-sensing gate in which active mTOR complex 1 (mTORC1) restrains autophagy and mTOR inhibition releases that brake; accordingly, rapamycin-driven mTOR suppression increases autophagosome formation and has been associated with accelerated or amplified NET output under stimulus-dependent conditions, likely because enhanced recycling and homeostatic buffering lower the threshold for executing NET-associated effector modules [57]. In contrast, tumor microenvironments characterized by high mTOR signaling can coincide with reduced NET production, a pattern that fits with autophagy suppression when mTOR tone is elevated. Importantly, PI3K signaling is not uniform across isoforms or contexts, and distinct PI3K nodes can differentially influence autophagy, NADPH oxidase activity, and ROS-linked NET pathways, helping explain why autophagy-targeting interventions yield heterogeneous outcomes [59]. This complexity is illustrated by 3-methyladenine (3-MA), which can decrease NET formation in disorders such as ANCA-associated vasculitis and leukemia, yet may not prevent rapamycin-enhanced NET release, consistent with 3-MA affecting multiple PI3K-dependent steps—including those that support NOX2-derived ROS generation—rather than acting as a selective, universal “autophagy-off” switch [60].

#### 2.1.5. Beyond Antimicrobial Defense: NETs as Mediators of Sterile Inflammation, Thrombosis, and Cancer Aggressiveness

Neutrophil extracellular traps were originally recognized as a host-defense strategy that helps contain infection by immobilizing pathogens in an extracellular chromatin scaffold enriched with antimicrobial proteins, thereby limiting dissemination and facilitating clearance by other immune mechanisms [11,61]. When appropriately controlled, this response contributes to tissue protection; however, excessive or persistent NET formation can become pathogenic by sustaining sterile inflammation and exposing immunogenic nuclear components [62]. In this setting, extracellular DNA and histones can function as danger-associated molecular patterns (DAMPs) and autoantigens, promoting chronic innate and adaptive immune activation that has been implicated across a spectrum of inflammatory phenotypes, including immune-complex-driven disorders and organ-damaging inflammatory states [62,63]. NET burden has also been linked to immunothrombotic responses, reflecting a broader interface between inflammation and coagulation in which NET scaffolds can provide a platform for platelet and coagulation factor engagement [46,64].

In cancer biology, NETs are increasingly viewed as active remodeling elements within the tumor ecosystem rather than passive byproducts of inflammation [65]. A consistent theme is that NET deposition amplifies pro-tumor inflammation and reshapes stromal architecture, creating conditions that favor tumor cell survival, invasion, and dissemination [11]. NET-associated proteases, particularly NE and MPO, can contribute to extracellular matrix (ECM) remodeling and altered cell–cell adhesion, processes that support epithelial–mesenchymal transition (EMT)-like phenotypes and enhance motility [66,67]. In parallel, NET-derived extracellular DNA can act directly on tumor cells as a bioactive signal and, in some settings, has been associated with increased invasiveness [65]. These local effects extend systemically: NET structures and their DNA–histone complexes can promote physical trapping and adhesion of circulating tumor cells (CTCs) to vascular and stromal surfaces, supported by adhesion receptor axes such as β1-integrin-dependent interactions, thereby increasing the probability of metastatic seeding [44,68].

NETs also influence anti-tumor immunity and therapy response by modulating immune cell access and function within the tumor microenvironment (TME) [69]. Dense NET networks can form a physical and biochemical barrier that limits effective contact between malignant cells and cytotoxic effector populations, including CD8^+^ T cells and natural killer (NK) cells, while simultaneously reinforcing immunosuppressive circuits through cytokine- and stromal-fibroblast-dependent reprogramming [44,70]. Moreover, NET-driven inflammatory cues integrate with platelet–endothelial–neutrophil crosstalk to promote a hypercoagulable state, providing a mechanistic link to cancer-associated thrombosis and perioperative metastasis susceptibility [71]. Together, these observations support a feed-forward model in which tumor-associated inflammation promotes NET release, and NETs, in turn, reinforce a microenvironment that is permissive for tumor progression, metastatic spread, immune escape, and treatment resistance.

## 3. NETosis in Acute Pancreatitis: Mechanistic Drivers, Biomarkers, and Clinical Consequences

Acute pancreatitis is an inflammatory injury of the pancreas initiated by inappropriate intrapancreatic activation of digestive zymogens, most notably trypsinogen, which drives acinar cell damage and local “autodigestion” with variable extension beyond the gland [72,73]. Epidemiological data suggest that acute pancreatitis affects approximately 34 individuals per 100,000 population each year, and while most episodes are mild and self-limiting, approximately 20–30% of patients progress to severe acute pancreatitis (SAP), a critical phenotype associated with infected necrosis, systemic inflammatory response syndrome (SIRS), and multiple organ failure, with reported mortality reaching 20–40% in high-risk cohorts [72,74]. Gallstones are the most common cause of acute pancreatitis, followed by excessive alcohol consumption [75]. Regardless of the underlying trigger, the progression from localized pancreatic injury to SAP is largely shaped by the host immune response [76]. Repeated or extensive damage to acinar cells leads to a surge in proinflammatory cytokines and chemokines, which recruit and activate innate immune cells [77]. While this response can aid in controlling infection and clearing damaged tissue, excessive or dysregulated activation may worsen tissue injury, drive pancreatic necrosis, and promote systemic inflammation and multi-organ dysfunction [78].

Neutrophils play a central role in amplifying inflammation in AP, especially in its severe form, where trypsin activation, leukocyte infiltration, and microvascular dysfunction synergistically worsen tissue injury [79,80]. Experimental studies have shown that neutrophil recruitment is a critical step in this process, as depletion of neutrophils or blockade of adhesion molecules such as P-selectin, lymphocyte function-associated antigen 1 (LFA-1; CD11a/CD18), and intercellular adhesion molecule 1 (ICAM-1; CD54) leads to reduced pancreatic damage [78]. Their migration into the inflamed pancreas is further guided by CXC chemokines (chemokines with two cysteines separated by one amino acid), such as C-X-C motif chemokine ligand 2 (CXCL2), and inhibition of these chemotactic pathways has been shown to attenuate injury, highlighting the causal contribution of neutrophil trafficking [81]. Once within the pancreatic tissue, activated neutrophils exacerbate injury by releasing ROS and proteolytic enzymes, including NE and matrix metalloproteinase 9 (MMP-9) [82]. Beyond these well-established cytotoxic mechanisms, neutrophils can also expel extracellular traps composed of DNA, histones, granule proteins, and alarmins such as high mobility group box 1 (HMGB1) [83]. These neutrophil extracellular traps can accumulate both within the pancreas and in the systemic circulation, where they further amplify inflammation and tissue damage during acute pancreatitis [84].

Neutrophil influx is a defining histopathologic feature of pancreatitis and directly contributes to parenchymal injury through oxidants and proteases [85]. In parallel, activated neutrophils release NETs that can further amplify pancreatic inflammation [86]. In SAP, this response becomes a “double-edged” mechanism: while NETs may support antimicrobial containment, excessive or dysregulated NET deposition can aggravate local tissue damage early in the disease course and has been linked experimentally to downstream complications, including microvascular thrombosis, secondary infection, sepsis, and multi-organ dysfunction [87,88].

### 3.1. Mainstream Framework of Acute Pancreatitis Initiation and Progression: Ca^2+^–ATP Failure, Necrosis, and Inflammatory Cell Invasion

Acute pancreatitis is now widely conceptualized as a disease in which the earliest, pancreas-intrinsic events set the stage for a subsequent self-amplifying inflammatory and microvascular cascade [89]. In the current mainstream framework, the initiating insult (most commonly biliary, alcohol-related, or metabolic) converges on pathological acinar-cell Ca^2+^ signaling, leading to mitochondrial dysfunction, impaired ATP generation, defective cellular homeostasis, and ultimately acinar cell death, with necrosis representing the decisive inflection point for severe disease [89,90]. This Ca^2+^–mitochondria–ATP axis provides a coherent explanation for why AP can rapidly transition from a local pancreatic injury to systemic illness, because necrotic acinar cells release danger signals that trigger robust innate immune activation and promote inflammatory cell invasion of the pancreas [89].

From this perspective, the most clinically dangerous aspect of AP—early and persistent organ failure—should be interpreted primarily as a consequence of systemic injury driven by extensive pancreatic necrosis and its downstream inflammatory and vascular sequelae, rather than as a direct readout of intra-acinar protease activation alone [78,79,91]. Organ failure may develop early as part of a sterile systemic inflammatory response, or later in association with infected necrosis and sepsis, and remains the dominant determinant of outcome [91].

Importantly, the inflammatory cell response in AP is not neutrophil-exclusive: macrophages are key early effectors that shape cytokine production, regulate tissue injury and repair programs, and interact bidirectionally with neutrophils during disease escalation [78]. Framing AP progression as a coordinated innate immune response (rather than a single-cell-type phenomenon) also helps reconcile biomarker and therapeutic observations across experimental models and human cohorts [92].

In parallel with cellular injury programs, AP is accompanied by a stereotyped inflammatory vascular response. Activation of the kallikrein–kinin system and bradykinin B2 receptor signaling has been implicated in pancreatic edema formation and hemodynamic consequences such as hemoconcentration, hypovolemia, and hypotension—physiological features that provide a mechanistic bridge between local pancreatic inflammation and systemic deterioration [93].

Within this established sequence, NET formation is best positioned as a propagation and amplification module rather than the primary initiating event. Once acinar necrosis and danger signaling recruit and prime innate immune cells, neutrophils can deploy NETs in pancreatic, peripancreatic, and systemic compartments, where NET-associated DNA–histone scaffolds and granule proteins may intensify DAMP-driven inflammation, promote microvascular dysfunction and immunothrombosis, and potentially contribute to duct-compartmentalized obstruction phenomena.

### 3.2. Circulating NET Biomarkers Track Severity in Human Acute Pancreatitis

In humans, multiple studies report that circulating NET burden increases in AP and tracks with clinical severity [87,94,95]. Compared with healthy controls, patients with AP, particularly those with SAP, show higher plasma and serum indices of NET formation, including elevated cfDNA, DNA–histone complexes, and more specific NET readouts such as CitH3 and MPO–DNA complexes [87,94,95,96]. These markers tend to rise early after hospital admission and increase stepwise with disease severity [87,94,95]. Importantly, higher NET-associated signatures have also been associated with septic complications, and CitH3 in particular has been reported to be enriched in septic AP compared with non-septic AP, with higher levels observed in patients requiring intensive care or experiencing fatal outcomes [96]. Collectively, these data support the presence of heightened NET activity in AP patient blood and link NET-associated biomarkers to severity stratification and sepsis risk, consistent with a pathobiological role for NETs in the human disease trajectory.

### 3.3. NETosis in Experimental Acute Pancreatitis: Initiation, Amplification Loops, and Resolution Pathways

In experimental pancreatitis, NET formation is readily detectable within injured pancreatic tissue and is coupled to systemic release of NET-associated products [87]. In the widely used sodium taurocholate model of acute necrotizing pancreatitis (ANP), taurocholate infusion drives extensive extracellular DNA deposition in the inflamed pancreas, which co-localizes with neutrophil granule constituents such as elastase and with histone markers (e.g., histone 2B), consistent with bona fide NET structures [87]. This local NET accumulation is accompanied by increased circulating cfDNA, indicating spillover into the systemic compartment [87]. In addition to reflecting neutrophil activation, these NET-rich deposits have been implicated in shaping inflammatory cell trafficking, including the recruitment of neutrophils within the pancreas and at distant sites such as the lung, aligning NET biology with both local pancreatic injury and extra-pancreatic inflammatory complications in severe disease [87].

Experimental work supports a bidirectional relationship between NET formation and neutrophil recruitment and activation in AP. When NET generation is inhibited, systemic inflammatory tone is attenuated, with reduced circulating levels of key mediators such as interleukin 6 (IL-6), HMGB1, CXCL2, and MMP-9, a protease implicated in pancreatic injury amplification [87]. Mechanistically, NETs appear to regulate neutrophil influx at two complementary levels: indirectly, by promoting pancreatic expression of the CXC chemokine CXCL2, which coordinates tissue navigation and acts as a strong stimulus for neutrophil activation; and directly, by upregulating the β2-integrin macrophage-1 antigen (Mac-1; CD11b/CD18) on circulating neutrophils, an adhesion receptor required for firm arrest and extravasation into inflamed tissue [87]. In line with this model, NET exposure enhances reactive ROS generation in isolated neutrophils, indicating that NETs can function as activating ligands that further prime neutrophils for inflammatory effector responses [97].

In addition to this NET–CXCL2–Mac-1 axis, recent findings suggest that dynamic changes in neutrophil surface markers are associated with the severity of acute pancreatitis and are closely linked to the regulation of NET formation. Cluster of differentiation 177 (CD177), a neutrophil-restricted glycoprotein (NB1) with high affinity for platelet endothelial cell adhesion molecule 1 (PECAM-1), has been associated with neutrophil transmigration and appears to track with disease severity [98]. Experimental blockade has been reported to mitigate caerulein-induced pancreatic injury and lung involvement, accompanied by reduced NET formation in vitro, potentially through dampening oxidative stress [98]. Separately, the tetraspanin CD53 (TSPAN25), a pan-leukocyte antigen expressed on multiple immune lineages including granulocytes, has been found upregulated in in vitro NET models and in neutrophils from AP patients and has been proposed to promote NET formation through phosphoinositide 3-kinase/protein kinase B (PI3K/AKT) signaling, a pathway capable of enhancing NADPH oxidase activity and ROS generation [99]. CD53 may also modulate adhesion programs via integrin-associated effects, thereby linking leukocyte aggregation at inflamed sites with NET-permissive signaling states [99].

Several upstream signals have been implicated in priming neutrophils for NET formation during AP. P-selectin (CD62P), which is rapidly translocated to the surface of activated platelets (from α-granules) and endothelial cells (from Weibel–Palade bodies), is increased in the circulation of AP patients at presentation and has been reported to correlate with clinical course [100,101]. Functionally, P-selectin can engage P-selectin glycoprotein ligand 1 (PSGL-1) on neutrophils, linking vascular activation to neutrophil priming and creating conditions that favor NET release [101]. At the signaling level, the P-selectin–PSGL-1 interaction is proposed to initiate an intracellular signaling cascade that converges on chromatin remodeling. PSGL-1 engagement promotes spleen tyrosine kinase (Syk) phosphorylation and mobilizes intracellular calcium (Ca^2+^) signals, including Ca^2+^ flux from endoplasmic reticulum (ER) stores into the cytosol [101]. Ca^2+^ is a key cofactor for PAD4, a Ca^2+^ regulated enzyme with multiple Ca2+-binding sites. Ca^2+^ binding induces conformational changes that enable PAD4 catalytic activity [101]. Activated PAD4 citrullinates histones, weakens histone–DNA interactions, and facilitates chromatin decondensation, thereby lowering the threshold for NET deployment. In this framework, P-selectin acts as an upstream amplifier of NET biology by driving a PSGL-1–Syk– Ca^2+^–PAD4 axis rather than merely promoting physical neutrophil tethering [101].

Terminal execution of NET release also appears to require membrane-permeabilization machinery. Another key molecular player with potential translational significance is GSDMD, a pore-forming effector best known for executing pyroptosis after cleavage by inflammatory caspases (classically caspase-1, and in nonclassical pathways caspase-11 in mice) [102]. In AP, increased GSDMD activity has been reported in neutrophils from experimental models and patients, and pharmacologic inhibition of GSDMD has been associated with reduced NET formation together with attenuation of pancreatic injury, systemic inflammation, and organ dysfunction in mouse models [103]. These observations support a model in which membrane permeabilization steps mediated by GSDMD contribute to NET release efficiency, while, in parallel, GSDMD-driven pyroptotic programs in pancreatic acinar cells may worsen necrosis and SIRS, reinforcing the inflammatory milieu that further promotes NET generation [103].

A complementary danger-signal pathway involves extracellular cold-inducible RNA-binding protein (eCIRP), a stress-released DAMP [104]. Under inflammatory stress, CIRP can relocate from its nuclear role in RNA regulation to the extracellular space, where eCIRP promotes endothelial activation, macrophage cytokine release, and NET formation. In severe AP models, blocking eCIRP has been associated with reduced pancreatic CitH3 and NET deposition, lower circulating DNA–histone complexes, and attenuation of chemokine production in the pancreas alongside decreased plasma IL-6, HMGB1, and MMP-9. Notably, eCIRP has been detected bound to NET structures and has been proposed to act as a NET-associated agonist capable of activating acinar cells, providing a feed-forward loop between neutrophil effector programs and pancreatic parenchymal activation. Clinically, circulating eCIRP has been reported to correlate with AP severity, supporting its candidacy as a biomarker and a mechanistic node connecting sterile danger signaling to NET-driven immunopathology.

Counter-regulatory circuits have also been described, consistent with the concept that NET output reflects the balance between pro-inflammatory induction and resolution pathways. Protectin D1 (PD1), a docosahexaenoic acid (DHA)-derived specialized pro-resolving mediator, has been reported to attenuate pancreatitis severity in mice while reducing pancreatic neutrophil infiltration and NET-related markers such as CitH3 [105]. Mechanistically, PD1 has been linked to lower PAD4 expression and reduced cfDNA/CitH3 release from neutrophils in vitro, suggesting that pro-resolving lipid mediators can constrain early neutrophil recruitment and suppress PAD4-dependent NET formation [105]. Together, these findings frame NETosis in AP as a regulated output shaped by adhesion-driven Ca^2+^–PAD4 signaling, pore-forming execution machinery, DAMP amplification loops, and endogenous resolution mediators that can break the response.

### 3.4. NET-Driven Pancreatic Injury in Acute Pancreatitis: Histone Signaling, Trypsin Activation, and Ductal Aggregation

Multiple lines of preclinical evidence point to a mechanistic connection between NET biology and the defining early event in AP, namely premature intra-acinar trypsinogen activation. Although early acinar cell dysfunction is classically associated with premature activation of digestive proteases, progression to systemic inflammation and organ failure appears to be more tightly related to acinar necrosis resulting from sustained intracellular calcium elevation and depletion of ATP [89]. This metabolic collapse promotes extensive activation of innate immune pathways and facilitates inflammatory cell infiltration, thereby amplifying local pancreatic injury into a systemic inflammatory response [89]. In taurocholate-driven disease models, pharmacologic interference with NET formation substantially reduces circulating matrix MMP-9, a neutrophil-derived protease that can facilitate trypsinogen activation and thereby intensify acinar injury [87]. NETs also appear to act directly on acinar cells: exposure of acinar cells to NET preparations increases trypsin activity in parallel with phosphorylation of signal transducer and activator of transcription 3 (STAT3), a central acinar stress-response hub [87,106]. Current evidence indicates that histones associated with neutrophil extracellular traps, including histone H2A, histone H2B, histone H3, and histone H4, are likely the primary mediators of this effect [87]. These histones, rather than the DNA scaffold alone, appear to constitute the main bioactive component of the traps [87]. As strongly cationic proteins, extracellular histones can bind to negatively charged components of cell membranes and activate danger-sensing pathways. Experimental studies in pancreatic acinar tumor cells have shown that histones, especially histone H4, can stimulate membrane-associated toll-like receptor 9 (TLR-9), induce calcium oscillations, and initiate stress signaling cascades that ultimately converge on STAT3 activation [87,107]. In this framework, STAT3 phosphorylation and intra-acinar protease activation rise as a coupled response to histone-driven danger signaling, supporting the view that NET products are not merely downstream markers of inflammation but can feed forward to reinforce trypsin-dependent autodigestion through coordinated MMP-9- and STAT3-linked mechanisms [87].

NETs can also injure the pancreas through direct cytotoxicity. NET-derived histones are broadly toxic to epithelial and endothelial cells, and similar effects have been described in acinar cells, where histones reduce viability and compromise membrane integrity [87,108]. This mechanism aligns with the concept that excessive NET deposition transforms a protective antimicrobial scaffold into a tissue-damaging effector. In practice, this creates a feed-forward loop: acinar injury recruits neutrophils, neutrophils generate NETs, and NET components further amplify acinar dysfunction, trypsin activation, and cell death [87].

A distinct, tissue-architectural mechanism involves intraductal NET aggregation. Under inflammatory conditions, neutrophils can enter the lumen of biliopancreatic ducts and release decondensed chromatin in a PAD4-dependent manner [109,110]. The resulting DNase-sensitive NET material, enriched with neutrophil serine proteases, can trap particulate matter, cellular debris, and microbes, but it also promotes the formation of dense NET aggregates [109]. These aggregates can physically occlude pancreatic ducts, impair secretory flow, and drive focal pancreatitis and parenchymal remodeling. Genetic disruption of PAD4 reduces intraductal NET formation and has been linked to protection from progression in these models, reinforcing a causal relationship between NET-mediated duct obstruction and sustained pancreatic inflammation [109,110].

At the same time, NET aggregation may exert a containment function in necrotizing disease. In areas of pancreatic necrosis, condensed layers of aggregated NETs can form a provisional barrier that spatially separates necrotic debris from adjacent viable tissue [111]. By binding DAMPs to the chromatin scaffold and exposing them to proteolytic activity within the NET matrix, these structures may limit diffusion of necrosis-derived inflammatory mediators and locally buffer the inflammatory burden [111,112]. This barrier-like organization can also interface with coagulation, as extracellular DNA and chromatin-bound proteins provide a pro-thrombotic surface that reinforces fibrin deposition [111]. Over time, the NET-based scaffold may be replaced by fibroblast ingrowth and fibrosis, suggesting a trajectory from acute containment to longer-term tissue remodeling [111].

### 3.5. Hypertriglyceridemic Pancreatitis: NET Amplification and Worse Clinical Trajectories

Hypertriglyceridemic pancreatitis (HTGP) is increasingly encountered and is often associated with a more complicated clinical course, including higher rates of infected pancreatic necrosis (IPN), organ failure, prolonged hospitalization, and increased mortality [65]. Recent studies suggest that this phenotype is closely associated with enhanced NET formation, which appears to be influenced by both the host’s inflammatory responses and metabolic signals originating from the gut [113]. Experimental studies have shown that NET formation driven by PAD4, along with an immune environment dominated by interleukin-17A, plays a major role in amplifying both local and systemic inflammation HTGP [113]. A gut microbiota axis appears to intersect with this pathway: microbiome manipulation alters NET-related immune outputs, and microbial production of taurine has been reported to restrain NET formation in the pancreas and circulation [113]. Taurine can directly limit NET release induced by strong agonists such PMA or hypochlorous acid, and it has been linked to suppression of MAPK signaling and disruption of NADPH oxidase activity, thereby reducing oxidant-driven NET programs and preserving neutrophil viability [113,114]. On the other hand, a decrease in bacteria-derived taurine may shift mucosal immunity toward a T helper 17 (Th17)-dominant profile, disrupting the balance between Th17 and regulatory T cells (Tregs) [113]. This imbalance leads to increased levels of interleukin-17A in the gut, a cytokine known to strongly promote NET formation [115]. Together, these observations support a model in which metabolic products of the gut microbiota modulate neutrophil recruitment and NET output in lipid-driven pancreatic injury, positioning NET regulation as a plausible mechanistic bridge between dysbiosis, IL-17A signaling, and adverse outcomes in HTGP. The key NET-linked mechanisms, circulating biomarkers, and translational implications across human and experimental acute pancreatitis are summarized in Table 1.

## 4. NETs in Pancreatic Malignancies: From Immune-Cold Stroma to Metastasis, Thrombosis, and Therapy Resistance

Cancer remains a major global health challenge and a leading cause of death. According to the most recent GLOBOCAN 2022 estimates, approximately 20.0 million new cancer cases and 9.7 million cancer-related deaths occurred worldwide in 2022 [116]. Demographic projections indicate that the annual number of new cancer cases will exceed 35 million by 2050, representing an approximate 77% increase compared with 2022 [116]. Among malignancies, pancreatic cancer accounted for roughly 511,000 new cases and 467,000 deaths globally in 2022, underscoring its marked lethality and the near-parity between incidence and mortality [116]. Pancreatic ductal adenocarcinoma represents the predominant histological subtype, comprising at least 90% of pancreatic tumors in population-based datasets, and long-term outcomes remain poor; international comparisons typically report 5-year survival ranging from 5% to 15%, depending on healthcare setting, stage distribution, and case mix [117].

Biologically, PDAC is often described as an immunologically “cold” tumor because effective anti-tumor T-cell responses are scarce and clinical benefit from checkpoint blockade remains minimal [118,119]. A growing literature links this immune-refractory phenotype to tumor-promoting inflammation and to NETs, which have been associated with accelerated disease progression and inferior outcomes [120,121]. PDAC is also defined by a dense desmoplastic stroma, arising in part from pancreatic stellate cell (PSC) activation that creates a fibrotic and mechanically stiff TME [122]. Together, inflammation that promotes tumor growth, along with dynamic remodeling of the stroma, creates a supportive microenvironment that facilitates malignant cell survival and proliferation [123]. At the same time, these changes contribute to immune evasion by fostering an immunosuppressive tumor microenvironment. Within these conditions, NET-induced tissue remodeling and immune modulation may further enhance the aggressive behavior of pancreatic ductal adenocarcinoma [124,125].

In PDAC, neutrophils are frequently over-recruited to the TME and, together with tumor-associated macrophages, comprise a dominant innate immune compartment that correlates with adverse clinical behavior [126,127]. Within the PDAC TME, neutrophils are activated by tumor- and stroma-derived cues and can support tumorigenesis by reinforcing a milieu that is simultaneously inflammatory and immunosuppressive, contributing to desmoplasia and impaired anti-tumor immunity [127]. Mechanistically, neutrophils have been implicated across the metastatic continuum, from promoting local invasion and intravasation to conditioning premetastatic niches, facilitating extravasation, and supporting outgrowth and recurrence, often through cooperative interactions with tumor cells, platelets, and other immune populations [128,129,130]. Clinically, systemic neutrophil skewing is captured by the neutrophil-to-lymphocyte ratio (NLR), where a high NLR is repeatedly associated with worse survival in pancreatic cancer [131]. Beyond their effects at the primary site, neutrophil effector programs, including NET formation, have been linked to metastatic competence, underscoring neutrophils as both biomarkers of aggressive disease and functional drivers of PDAC progression [132].

### 4.1. Experimental PDAC Evidence for NET Amplification Across Tumor and Systemic Compartments

Experimental PDAC models consistently show heightened NET activity within the tumor-bearing host. In orthotopic murine pancreatic cancer, NET abundance is increased in tumor tissue and systemic compartments, supporting the concept that PDAC promotes a NET-permissive inflammatory state [120,133]. From a mechanistic perspective, pancreatic cancer cells (PaCa cells) can directly trigger rapid NET release from neutrophils, and this response has been reported to occur even when classical ROS-dependent programs are not dominant, suggesting that PDAC can activate alternative signals that promote NET formation [120]. In addition to malignant cells, other elements of the tumor microenvironment also contribute to enhanced NET formation. Cancer-associated fibroblasts (CAFs) and activated platelets have been identified as important sources of NET-inducing signals [134,135,136]. Together, these components help sustain a multicellular feed-forward loop, in which interactions between tumor cells, stromal elements, and the vasculature amplify neutrophil activation and promote continued NET release during the progression of pancreatic ductal adenocarcinoma [134].

### 4.2. Clinical Evidence Linking NET Activity to PDAC Aggressiveness and Recurrence Risk

Clinically, patients with PDAC frequently exhibit elevated circulating markers of NETs, supporting the concept that NET activity is not confined to the tumor bed but is reflected systemically and may participate in disease biology [137]. In PDAC, elevated levels of NET markers in plasma or serum are frequently observed [138]. In parallel, studies have also reported impaired NET clearance, suggesting that the delicate balance between NET formation and degradation is disrupted, favoring their persistence in the circulation and tumor microenvironment [138]. Consistent with this, analyses of patient-derived samples have shown that neutrophils from individuals with PDAC exhibit increased NET formation [120,133,139]. In addition, tumor tissue sections reveal a higher abundance of NETs compared to adjacent non-tumor areas, indicating that NET production is enhanced both systemically and within the tumor microenvironment [139].

Importantly, NET burden has been linked to clinical outcomes. Multiple cohorts report that increased NETs, particularly tumor-infiltrating NETs, associate with shorter overall survival (OS) and reduced recurrence-free survival (RFS), and NET measures have been proposed as independent prognostic variables rather than mere correlates of advanced stage [140]. In a large surgical series of resected PDAC, tumor NET levels predicted worse postsurgical OS and RFS, and multivariable modeling identified NETs alongside AJCC TNM stage as independent determinants of outcome [140]. Integrating NET assessment with standard TNM staging improved risk stratification compared with staging alone, underscoring the potential value of NET-related pathology as a clinically actionable biomarker of aggressive tumor behavior and recurrence propensity [140].

Major surgical procedures can trigger a systemic inflammatory response, partly as a result of tissue injury and ischemia followed by reperfusion [141]. This inflammatory state creates conditions that favor neutrophil activation and the release of NETs. In the context of PDAC, such perioperative immune dynamics may have clinical significance, as they can influence the tumor microenvironment and potentially affect recurrence risk and overall survival [140]. In various types of cancer, transcriptomic analyses based on gene expression profiles related to NETs have shown that higher NET activity scores are associated with worse clinical outcomes. This pattern has also been observed in pancreatic cancer, supporting the notion that tumors enriched with NET-related signals tend to exhibit more aggressive behavior [142]. In the perioperative setting, clinical studies of pancreatectomy have shown that circulating NET markers such as cfDNA and CitH3 rise after resection and peak several days postoperatively, consistent with surgery-induced cytokine surges that include NET-promoting mediators like interleukin 8 (IL-8), granulocyte colony-stimulating factor (G-CSF), and IL-6 [143]. Importantly, higher or persistent postoperative NET signatures have been linked with complications, and sustained elevation has been reported in association with pancreatic leak, suggesting that prolonged inflammatory signaling can maintain NET activity beyond the immediate postoperative window and potentially shape recovery and oncologic risk trajectories [143].

### 4.3. Tumor-Intrinsic Drivers of NETosis in PDAC: Epigenetic Rewiring and Soluble Signaling Axes

Epigenetic changes within pancreatic cancer cells can actively shape the tumor microenvironment in ways that promote neutrophil infiltration and enhance NET accumulation. Lysine-specific demethylase 6A (KDM6A), also known as UTX, is an X-linked enzyme that removes the repressive H3K27me3 histone mark and participates in transcriptional control programs important for pancreatic cell identity [144,145]. In PDAC, KDM6A is frequently inactivated and functions as a tumor suppressor, with loss linked to more aggressive behavior and adverse prognosis [146]. In terms of underlying mechanisms, KDM6A-deficient PDAC cells upregulate chemotactic cytokines, most prominently CXC motif chemokine ligand 1 (CXCL1), a well-established neutrophil-recruiting chemokine that signals through C-X-C chemokine receptor 2 (CXCR2) [147]. Increased CXCL1 secretion enhances tumor-associated neutrophil (TAN) accumulation and has been associated with higher NET formation [147]. In preclinical models, neutralizing CXCL1 reduces neutrophil chemotaxis and attenuates the NET-promoting activity of KDM6A-deficient tumor cells, with concomitant suppression of tumor growth, supporting the CXCL1–CXCR2 axis as a tractable vulnerability in low-KDM6A PDAC [147].

A second tumor-intrinsic route to NET induction involves soluble mediators that directly activate neutrophils. Tumor-derived tissue inhibitor of metalloproteinases 1 (TIMP1) exemplifies this mechanism by acting beyond its classical role in matrix regulation and functioning as a cytokine-like ligand [148]. In PDAC tissue, elevated levels of TIMP1 have been associated with gene expression patterns indicating neutrophil activation [148]. Moreover, NET structures are often found in close proximity to areas with high TIMP1 expression, suggesting a spatial link between TIMP1 abundance and NET accumulation [148]. The proposed causal pathway is that TIMP1 engages the tetraspanin CD63 on neutrophils, which organizes a membrane signaling platform that transduces signals into the MAPK cascade, leading to MEK/ERK activation [149]. ERK activity can facilitate a NET-permissive intracellular state by promoting oxidant-dependent programs and chromatin remodeling, thereby increasing the probability of extracellular chromatin release decorated with granule proteins [11]. Functionally, experimental disruption of TIMP1 or NET formation improves outcomes in PDAC models, and circulating TIMP1 has been reported to correlate with NET-associated biomarkers, suggesting that this axis is measurable systemically [149]. Clinically, combining TIMP1 and NET readouts with CA19-9 has been proposed to refine prognostic stratification, and TIMP1 has also been explored as part of biomarker approaches for earlier detection in familial PDAC risk contexts [149].

### 4.4. NETs as Active Effectors in PDAC Progression

An induced formation of NETs in the pancreatic tumor microenvironment has been associated with the development and the progression of PDAC, involving several molecules and mechanisms. Functionally, exposure to NETs can directly promote more aggressive behavior in pancreatic cancer cells [139]. Experimental studies have shown that NET-rich environments enhance their ability to migrate and invade surrounding tissue in vitro [139]. These reciprocal interactions between NETs and tumor cells may further sustain inflammatory pathways that support metastasis. NETs also support tumor vascularization and can promote epithelial–mesenchymal transition (EMT), in part through NET-associated interleukin 1 beta (IL-1β) signaling that engages epidermal growth factor receptor (EGFR) and downstream ERK, thereby shifting tumor cells toward a more motile, invasive phenotype [150,151,152]. Beyond tumor-cell-intrinsic effects, NETs can remodel the stromal compartment by activating pancreatic stellate cells (PSCs), contributing to desmoplasia that protects tumor cells and limits immune penetration; related stellate-cell programs may also facilitate hepatic micrometastatic seeding [153,154]. Based on current understanding of the mechanism, these pro-aggressive effects have been linked to convergent inflammatory axes, including IL-1β–EGFR–ERK signaling, receptor for advanced glycation end products (RAGE)-dependent pathways, and PAD4-associated NET programs that intersect with IL-17-skewed immunoregulation, together reinforcing an immunosuppressive, metastasis-permissive niche in PDAC [138,152,153].

### 4.5. NET-Mediated Immune Remodeling in PDAC

Inflammation and NET formation appear to reinforce each other in PDAC, creating a self-sustaining loop that favors tumor progression. Inflammatory signals can prime neutrophils to release NETs, while the components released from NETs further aggravate local tissue damage and activate innate immune pathways [155]. This bidirectional interaction helps sustain a chronic inflammatory state that supports tumor progression [155]. This reciprocity is clinically relevant because cytokine networks that normally support host defense can be hijacked by cancer cells to remodel the TME toward immune escape and metastatic competence [156]. Among these mediators, interleukin 17 (IL-17) has emerged as a key driver linking inflammation to NETosis in PDAC [138].

Mechanistic studies indicate IL-17, produced by Th17 cells, accumulates within pancreatic cancer tissue and signals through the IL-17 receptor pathway. This signaling promotes the activation of both tumor and stromal cells, leading to the production of factors that attract neutrophils to the tumor site [157,158]. This recruitment is accompanied by PAD4-dependent NET formation, and the accumulation of NETs within the tumor can reshape the spatial distribution of immune cells, altering the immune landscape of the tumor microenvironment [138]. A consistent observation is that NETs play a role in excluding cytotoxic CD8^+^ T cells from the tumor core [138]. By forming both a physical and functional barrier, NETs hinder effective immune surveillance and help maintain an immunosuppressive microenvironment that supports tumor progression [138]. Importantly, blocking IL-17 signaling, either genetically or with pharmacologic agents, has been shown to reduce neutrophil infiltration and NET formation. This intervention can also restore responsiveness to immune checkpoint inhibitors in a manner that depends on CD8^+^ T cells, highlighting the IL-17-driven NET response as a potential and targetable mechanism of immune resistance [138]. Other inflammatory stimuli may lead to similar outcomes. For instance, PAF has been shown to enhance the tendency of neutrophils in pancreatic cancer to form NETs [120]. This is accompanied by increased levels of circulating DNA and citrullinated histone H3 in both experimental models and patient samples, reflecting a systemic inflammatory profile consistent with NET activation [120].

Chemokine signaling networks play a central role in maintaining neutrophil recruitment and driving sustained NET formation in PDAC. In collagen-rich tumors, collagen activation of discoid domain receptor 1 (DDR1) on cancer cells can induce CXCL5 production through NF-κB-linked signaling, promoting recruitment of tumor-associated neutrophils and NET formation that supports invasion and metastasis [159]. A second feed-forward loop is driven by IL-8, also known as C-X-C motif chemokine ligand 8 (CXCL8), a canonical neutrophil chemokine with additional pro-angiogenic effects [160]. Here, NET material can directly stimulate PDAC cells by delivering extracellular DNA that is sensed as dangerous, often together with histones and proteases. This signal activates the STING pathway, leading to TANK-binding kinase 1 (TBK1) phosphorylation and NF-κB activation, which increases IL-8/CXCL8 transcription and secretion [139,161,162]. Blocking STING reduces p-TBK1, lowers NF-κB activity, and decreases IL-8 output [163]. Tumor-derived IL-8 then recruits more neutrophils into the TME. When IL-8 levels rise locally, it can also activate neutrophils and promote NETosis [139]. This occurs through MAPK signaling, with increased phosphorylation of mitogen-activated protein kinase kinase (MEK) and extracellular signal-regulated kinase 1/2 (ERK1/2). Reactive oxygen species act as a key execution node, because MEK inhibition or ROS blockade suppresses NET release [164].

### 4.6. Linking NET Biology to Fibrosis in PDAC

PDAC is defined by a prominent desmoplastic reaction in which PSCs and CAFs generate a dense ECM rich in collagen that supports tumor progression and correlates with adverse prognosis [127]. NET biology interacts with fibrotic pathways on multiple levels. The DNA released through neutrophil extracellular traps can function as a bioactive ligand for the RAGE on pancreatic stellate cells [120]. This interaction promotes stellate cell activation and contributes to the expansion of a collagen-rich stroma [120]. Preclinical studies have demonstrated that DNA released from NETs can activate pancreatic stellate cells, thereby promoting tumor growth. These findings position NETs not merely as passive markers of inflammation, but as active contributors to stromal remodeling in pancreatic cancer [120]. A parallel line of communication between CAFs and NETs has also been described, in which fibroblasts help establish a microenvironment that favors NET formation [136]. They do so by recruiting neutrophils and triggering NETosis through redox-sensitive signals [136]. Amyloid-β produced by cancer-associated fibroblasts has been identified as a potential mediator of this process. More broadly, these findings suggest that fibroblast-driven programs can actively enhance NET formation within the pancreatic cancer microenvironment [136].

Another fibrosis-associated pathway links collagen signaling to neutrophil recruitment and NET formation. This circuit involves discoid domain receptor 1 (DDR1), a receptor tyrosine kinase activated by collagen, which is highly expressed in pancreatic ductal adenocarcinoma and has been associated with more invasive tumor behavior [165,166]. Collagen deposition can activate DDR1 on cancer cells and drive chemokine output, particularly CXCL5, which recruits tumor-associated neutrophils and promotes NET formation [159]. In terms of underlying mechanisms, CXCL5 induction has been linked to DDR1-dependent signaling that converges on NF-κB through intermediates such as protein kinase C theta (PKCθ) and spleen tyrosine kinase (SYK) [159]. This sequence establishes a self-reinforcing loop. The collagen-rich extracellular matrix activates DDR1, which in turn promotes the expression of CXCL5. CXCL5 attracts neutrophils and promotes NET formation, while the resulting NETs contribute to tumor invasion and metastasis by remodeling the inflammatory microenvironment [159].

### 4.7. Autophagy-Enabled NETosis in PDAC

In PDAC, NET formation appears to be coupled to a RAGE-driven autophagy program that links danger sensing to neutrophil effector remodeling within the tumor microenvironment [18]. The receptor for advanced glycation end products, a DNA- and DAMP-responsive receptor implicated in pancreatic carcinogenesis and myeloid immunosuppression, can promote inflammatory signaling and autophagic activity [120]. In neutrophils, this autophagy induction is permissive for NET release [120]. In neutrophils, engagement of RAGE by PDAC-enriched DAMPs such as HMGB1 induces autophagic flux that supports the intracellular trafficking and metabolic/redox capacity required for NETosis, thereby creating a permissive state for NET release [120]. In this context, autophagy supports the mobilization of granule enzymes and enhances the cell’s ability to generate ROS. At the same time, PAD4 promotes chromatin decondensation by catalyzing histone citrullination, leading to the formation of CitH3. This process allows the chromatin, now coated with neutrophil-derived proteases, to be released into the extracellular space as part of NET formation [120]. In orthotopic PDAC models, tumor-bearing hosts show an increased propensity for NET formation that is reduced by genetic ablation of RAGE or pharmacologic blockade of autophagy (e.g., chloroquine), accompanied by lower circulating DNA and CitH3 signals [120]. Consistent with a functional role, the absence of PAD4 has been shown to slow pancreatic tumor growth, reduce activation of stromal components, lower levels of extracellular DNA in the bloodstream, and improve overall survival. These findings support the idea that a signaling axis involving the RAGE, autophagy, and PAD4 sustains ongoing NET formation. In turn, NETs contribute to fibrotic remodeling, tumor progression, and the metastatic potential of pancreatic ductal adenocarcinoma [120].

### 4.8. NET-Driven Metastatic Seeding in PDAC

NETs have been detected in close proximity to metastatic deposits and can be induced systemically by PDAC, suggesting that NETs actively support the metastatic process, rather than simply marking inflammation at distant sites. In terms of underlying mechanisms, NET structures can physically capture circulating pancreatic cancer cells, increasing their adhesion to the vascular wall and supporting subsequent transendothelial passage, thereby strengthening the extravasation step [151]. Experimental data further suggest that “pre-existing” NETs within metastatic organs, particularly the liver, create a permissive niche before overt metastases form [167]. In mouse models, NET formation in the liver has been observed even before metastatic lesions become detectable. This early accumulation of NETs appears to create a microenvironment that attracts circulating tumor cells and promotes their adhesion, effectively preparing the liver for metastatic seeding [167]. Beyond this structural function, NET-derived extracellular DNA can act as a signaling ligand that amplifies pro-metastatic inflammation in PDAC, including induction of chemokines such as CXCL8, which can further promote tumor cell motility, invasion, and colonization [168].

### 4.9. NETs as EMT Triggers in PDAC

Epithelial–mesenchymal transition (EMT) is a central enabling program for dissemination in PDAC because it reduces epithelial cohesion and equips tumor cells with motility and invasive capacity [169]. Experimental work indicates that NET exposure can push pancreatic cancer cells toward this phenotype [139]. In vitro, NET-treated PANC-1 and MIAPaCa-2 cells show a marker shift consistent with EMT, with reduced E-cadherin and increased mesenchymal features such as N-cadherin, vimentin, and α-smooth muscle actin (α-SMA), accompanied by higher migratory and invasive behavior [139]. Clinical relevance is supported by observations that neutrophils from PDAC patients can directly promote tumor cell motility, and that NETs generated from either patient or healthy-donor neutrophils can reproduce this pro-migratory, pro-invasive effect [152]. Taken together, these findings suggest that NETs do more than simply serve as a structural framework. They actively influence tumor cell behavior by triggering signaling pathways that promote a state favorable for metastasis.

Several convergent signaling routes plausibly explain how NETs couple inflammation to EMT. NETosis releases cytokines and alarmins that can engage tumor cell receptors and activate canonical EMT pathways [151,170]. A prominent example is NET-associated interleukin 1 beta (IL-1β), which can stimulate EGFR/ERK signaling and has been implicated as a necessary mediator of NET-driven migration, invasion, and EMT in PDAC models [152]. This signaling axis is biologically plausible, as IL-1β is a strong inducer of inflammatory gene expression and can interact with key EMT regulators by engaging in crosstalk with pathways such as transforming growth factor beta (TGF-β) signaling [171]. In addition, HMGB1 present on NETs can function as a paracrine EMT trigger [170]. PDAC cells express multiple HMGB1-responsive receptors, including TLR2, TLR4, RAGE, and CD24, allowing NET-derived HMGB1 to activate downstream MAPK/ERK and NF-κB signaling and to modulate EMT transcriptional regulators such as SNAIL, SLUG, and ZEB1 [151,152,172]. In line with this, interventions that degrade or neutralize NET-associated HMGB1 have been reported to blunt malignant traits and reduce liver metastatic spread in experimental settings, underscoring the importance of NET cargo as an active EMT-inducing stimulus.

Beyond soluble mediators, DNA released from NETs can act as a pro-metastatic signal by engaging a specific receptor-to-cytoskeleton signaling pathway that guides tumor cell behavior and supports metastatic dissemination. Modified NET-DNA can bind the tumor cell surface protein coiled-coil domain-containing protein 25 (CCDC25), which then recruits integrin-linked kinase (ILK) and cytoskeletal adaptors such as β-parvin (PARVB) [167,173]. This CCDC25–integrin β1 (ITGB1)–ILK module reshapes cytoskeletal dynamics and promotes EMT-associated transcriptional programs [174]. Downstream of receptor activation, ILK promotes phosphorylation of AKT and GSK3β, which helps stabilize key regulators of epithelial-to-mesenchymal transition. This process also enhances β-catenin-dependent gene expression and supports tumor cell growth and survival by activating the mTOR pathway [175,176,177]. Notably, NET-related expression signatures that include components of this axis have been associated with poor outcomes, consistent with a model in which NET sensing at the tumor cell surface is translated into cytoskeletal remodeling, EMT activation, and metastatic competence [173]. Parallel tumor-intrinsic programs can intensify this vulnerability; for example, loss of the epigenetic regulator KDM6A has been linked to more aggressive, EMT-prone PDAC states and persistent inflammatory remodeling, potentially creating a context in which NET-driven EMT signals are more readily executed [147].

### 4.10. Antitumor NET Programs in PDAC

Although NETs are most often discussed as tumor-promoting structures in PDAC, selected contexts indicate that they can also support antitumor activity, emphasizing that biological impact depends on neutrophil state and the way NETs are generated. In a pancreatic cancer model, melatonin was reported to reprogram tumor–myeloid communication by inducing cancer cells to release the chemokine CXCL2, which recruits TANs and favors an N1-like, more cytotoxic phenotype [178]. Under these conditions, neutrophils underwent ROS-dependent NETosis, and the resulting oxidant-rich NET program was associated with enhanced tumor cell apoptosis, suggesting that NETs can participate in tumor control when coupled to a pro-inflammatory, antitumor neutrophil polarization [178]. At the same time, pancreatic ductal adenocarcinoma can take advantage of neutrophil-derived molecules such as MMP-9 and immunosuppressive enzymes like indoleamine 2,3-dioxygenase (IDO) to weaken NK cell activity [179]. This highlights the dual nature of NET-associated components, which can either support immune-mediated tumor elimination or be redirected toward immune suppression, depending on the prevailing inflammatory signals within the tumor microenvironment.

### 4.11. Immunothrombosis in PDAC: NETs as Structural and Biochemical Catalysts of Coagulation

Tumor-associated hypercoagulability is a defining systemic complication of PDAC and a major driver of venous thromboembolism (VTE), with clinically meaningful consequences for both quality of life and survival [180]. In PDAC, several prothrombotic signals originating from both the tumor and the host converge to promote a hypercoagulable state. These include elevated tissue factor (TF) activity, the release of tumor-derived microvesicles, inflammatory activation of various leukocyte populations, and the formation of NETs [181,182,183]. In this setting, NETs function as more than an inflammation marker; they provide a structural and biochemical platform that accelerates coagulation [133,135,150]. Clinical observations support this link, showing that NET burden in PDAC associates with higher-stage disease and measurable procoagulant activity, consistent with a model in which inflammation, endothelial activation, and NETosis jointly establish a thrombosis-prone intravascular milieu [133].

Mechanistically, NETs can shift endothelial cells (ECs) toward a procoagulant phenotype [133]. Exposure to NETs has been associated with EC dysfunction and morphological remodeling, including retraction at cell–cell junctions, which may facilitate vascular leak and prothrombotic surface remodeling [133]. At the biochemical level, NETs promote phosphatidylserine (PS) exposure and provide a scaffold for coagulation factor binding, thereby enhancing factor Xa (FXa) generation, thrombin (FIIa) production, and fibrin formation [133]. In parallel, NETs directly engage platelets: the DNA–histone meshwork supports platelet adhesion and aggregation, while abundant NET-associated histones can activate platelets and amplify thrombin generation [184,185,186]. Additional NET components, such as cathepsin G, can further promote platelet aggregation [187]. These effects on endothelial cells and platelets help explain why blood enriched with NETs exhibits heightened reactivity, leading to accelerated clot formation and an increased risk of thrombosis.

Experimental data reinforce causality in PDAC-associated thrombosis. In both xenograft and orthotopic models, mice with human pancreatic tumors display elevated levels of NET markers. When neutrophils are depleted or extracellular DNA is degraded using DNase I, the formation of venous thrombi is significantly reduced, indicating that intact NET structures contribute to thrombus formation [188]. PDAC cells and their conditioned media can also drive NET formation and increase endogenous thrombin generation, linking tumor-derived signals to systemic coagulation [150]. Importantly, disrupting key components of the NET formation pathway has been shown to reduce thrombosis in vivo. Genetic deletion of PAD4, a critical enzyme involved in NET release, prevents NET formation and significantly limits the development of thrombotic complications in tumor-bearing animals [189]. These findings align with clinical and translational observations suggesting that NETosis in PDAC is often sustained by RAGE- and autophagy-linked programs in neutrophils, and that pharmacologic inhibitors of these pathways can reduce perioperative VTE in treated cohorts [189]. More broadly, platelet–neutrophil cooperation offers an additional amplification loop: platelet activation can promote neutrophil activation and NET release through innate sensing pathways, creating a feed-forward circuit that couples inflammation to thrombosis and helps explain why PDAC is among the malignancies with the most pronounced thrombotic risk [46].

### 4.12. NET Biology Beyond PDAC: Emerging Roles in Pancreatic Neuroendocrine Tumors

Beyond PDAC, NET biology is increasingly recognized in other pancreatic malignancies, particularly pancreatic neuroendocrine tumors (pNETs), which represent the second most common malignant neoplasm of the pancreas and appear to be rising in incidence [190,191]. In pNET cohorts, the detection of intratumoral NETs has been linked to shorter recurrence-free survival (RFS), and both neutrophil infiltration and NET positivity have been reported as independent predictors of recurrence risk [191]. These observations fit within a broader mechanistic framework in which NET formation reflects coordinated crosstalk among neutrophils, tumor cells, platelets, and endothelial cells—an interactive network that can support tumor progression, dissemination, and a prothrombotic phenotype [192]. Functionally, NETs may facilitate malignancy by shaping tumor-cell programs (including stress and innate sensing pathways such as Toll-like receptor signaling and mitochondrial remodeling), by providing adhesive scaffolds that aid tumor cell arrest and extravasation, and by restructuring local immunity through physical and biochemical exclusion of cytotoxic CD8^+^ T cells and natural killer NK cells [167,193]. NETs can also influence stromal behavior by modulating tumor-associated fibroblasts, further reinforcing an immune-resistant, progression-permissive microenvironment in pancreatic neuroendocrine disease [153].

### 4.13. ChemoNETosis in PDAC: Neutrophil Dominance, ECM Remodeling, and Immune Escape

Gemcitabine-based regimens, including gemcitabine plus nab-paclitaxel (GnP), can unintentionally reprogram the pancreatic TME toward neutrophil dominance [194]. In mouse models of pancreatic cancer, treatment with gemcitabine and GnP has been associated with enhanced neutrophil infiltration and increased NET formation within the tumor. This therapy-induced inflammatory environment may reduce the overall effectiveness of treatment by creating conditions that support tumor resistance [194]. A major factor contributing to this effect is the surge in cytokines and chemokines following chemotherapy, particularly interleukin-8 (IL-8 or CXCL8). IL-8 serves as a strong chemoattractant for neutrophils and a potent inducer of NET formation, linking treatment-induced inflammation to changes in the tumor microenvironment [194,195]. In terms of underlying mechanisms, gemcitabine has been shown to trigger stress and inflammatory signaling pathways within cancer cells, often involving activation of NF-κB and STAT3. This leads to increased production of IL-8, which then acts on neutrophils through CXCR1 and CXCR2 receptors to stimulate NET formation—a process referred to as chemoNETosis [196]. Conceptually, this converts a cytotoxic intervention into a feed-forward inflammatory circuit: more IL-8 brings more neutrophils, and more neutrophils generate more NETs, progressively reinforcing a therapy-resistant TME.

At the level of tumor cells, multiple studies have demonstrated a direct link between NET-derived components and the development of resistance to therapy. In the context of gemcitabine and GnP treatment, pancreatic cancer cells have been shown to upregulate G protein-coupled receptor class C group 5 member A (GPRC5A), a molecule whose role in cancer is context-dependent but consistently associated with chemoresistance in pancreatic cancer [194]. Elevated GPRC5A expression enhances the secretion of IL-8, which further stimulates NET formation, at least in part by activating NLRP3 signaling in neutrophils [194,197]. Once released, NETs can support tumor survival and progression through multiple, overlapping mechanisms. The cell-free DNA within NETs can act as a signal that enhances cancer cell proliferation and motility [152]. At the same time, proteases associated with NETs, such as neutrophil elastase, MMP-9, and cathepsin G, can degrade and remodel the extracellular matrix, creating a microenvironment that favors tumor invasion and may contribute to the development of drug tolerance [198,199,200]. NET exposure has also been linked to pro-survival signaling in PDAC cells (including ERK pathway engagement) with downstream shifts in apoptotic control (e.g., Bcl-2 family balance) [196]. Finally, NETs may deepen immunosuppression, potentially via PD-L1 enrichment and by reinforcing CXCR2-skewed neutrophil activity, creating a vicious cycle in which resistant tumor clones further intensify NET induction, and NETs, in turn, sustain resistance and progression [201]. To provide an integrated overview, Table 2 summarizes the key NET-driven mechanisms described across pancreatic malignancies and highlights their potential clinical significance and therapeutic implication.

## 5. Therapeutic Targeting of NETs in Pancreatic Disease

Therapeutic inhibition of NETs has been explored most directly in AP, where excessive NET formation amplifies local injury and systemic inflammation [87]. A direct approach to limiting NET-mediated damage is to break down the NET scaffold. Deoxyribonuclease I (DNase I) degrades extracellular DNA, thereby disrupting the structural framework of NETs. In experimental models of SAP, DNase I treatment has been associated with reduced pancreatic edema, decreased deposition of histones within pancreatic tissue, and improvement in biochemical markers of disease severity [87]. Conceptually, this strategy is designed to target the structural framework of NETs rather than the upstream signals that activate neutrophils. By dismantling the extracellular DNA backbone, it may quickly reduce the harmful effects of NETs, including microvascular obstruction, local delivery of proteases, and the amplification of tissue injury within inflamed pancreatic tissue [87].

Another AP-oriented approach is to prevent NET generation by interrupting permissive intracellular programs. Since chromatin decondensation relies heavily on histone citrullination by PAD4, particularly the generation of CitH3, as well as the coordinated action of MPO and NE, targeting these pathways can effectively reduce NET formation [202]. Agents that disrupt PAD4 activity or interfere with MPO–NE function have been shown to suppress NET release by impairing key steps in the decondensation process. Chloroquine (CQ), classically used as an antimalarial and widely studied as an autophagy inhibitor, has been linked in murine SAP models to lower cfDNA and CitH3, reduced pancreatic injury, and attenuation of systemic mediators such as IL-6 and HMGB1 [96]. Although CQ has been proposed to limit intrapancreatic zymogen activation by interfering with lysosomal function, a NET-focused interpretation suggests a different mechanism. In this context, autophagy is considered a supportive process that enables NET formation. Inhibiting autophagy with agents like CQ reduces the ability of neutrophils to release chromatin in response to inflammatory stress, thereby limiting NETosis [96,120,203,204].

A complementary experimental direction in AP has focused on blocking the MPO–NE axis and oxidative amplification that supports NET release. Epigallocatechin-3-gallate (EGCG) has been reported to suppress NET formation in vivo, consistent with inhibition of NE activity and interference with MPO-dependent chromatin remodeling [205]. In these models, a decrease in NET formation is associated with less pancreatic tissue injury and a milder systemic inflammatory response. This includes lower levels of cytokines and chemokines that play a central role in attracting and activating neutrophils, suggesting that limiting NET burden may help interrupt key inflammatory circuits in acute pancreatic injury [205]. Taken together, findings from AP studies support a practical therapeutic approach. One option is to break down NETs after they are formed using agents like DNase. Alternatively, therapies can focus on preventing NET release by targeting upstream processes required for their formation, such as autophagy, protease activity, and oxidative signaling. Agents like CQ, EGCG, and related compounds have shown promise in this regard.

In PDAC, the rationale for NET-targeted therapy is broader and more mechanistically diverse, because NETs can reinforce immunosuppression, desmoplasia, metastatic seeding, and cancer-associated thrombosis [206]. One clinically familiar candidate is thrombomodulin (TM), an anticoagulant cofactor that binds thrombin and supports protein C activation but also exhibits anti-inflammatory activity against DAMPs [151]. In a metastasis-focused PDAC model, TM was proposed to reduce NET-driven dissemination by neutralizing extracellular HMGB1, a NET-associated alarmin that can promote EMT, inflammatory signaling, and metastatic competence once released into the extracellular space [151]. This framework is conceptually attractive in PDAC because HMGB1 has context-dependent biology: intracellular HMGB1 may support genomic stability and restrain early oncogenic programs, whereas extracellular HMGB1 can intensify inflammation and tissue remodeling that favor invasion and metastasis [172,207]. TM, in the presence of thrombin, has been described as capable of degrading HMGB1, thereby weakening HMGB1-dependent pro-metastatic signaling while simultaneously limiting NET-associated inflammatory propagation [151].

Chloroquine has also been positioned as a NET-directed agent in PDAC, particularly in the setting of hypercoagulability [189]. In orthotopic tumor models and patient-derived observations, inhibition of autophagy with CQ has been associated with reduced NET markers (serum DNA, CitH3) and attenuation of platelet activation/aggregation and procoagulant signals such as circulating tissue factor [189]. This aligns with the idea that RAGE-driven autophagy is permissive for NET formation in the PDAC milieu, and that reducing NETosis can, in turn, weaken the thrombo-inflammatory loop that links tumor biology to venous thromboembolism risk [120]. More broadly, any strategy that lowers NET burden may also reduce endothelial activation and the availability of NET scaffolds that support coagulation factor binding and fibrin generation.

In addition to agents like TM and CQ, several other drug classes have been shown to interact with NET-related pathways and may be considered as potential NET-modulating therapies in pancreatic ductal adenocarcinoma. Agents that limit platelet activation can indirectly suppress platelet–neutrophil cooperation, which is often required for robust NET induction in thrombo-inflammatory settings; acetylsalicylic acid (ASA) is one example, acting through cyclooxygenase (COX) inhibition and reduced thromboxane A2 signaling [208]. Classical anticoagulants may help mitigate NET-driven amplification of thrombosis by targeting key steps in the coagulation cascade. They can interfere with thrombin-dependent mechanisms that stabilize fibrin-rich clots on NET scaffolds and also limit downstream propagation of coagulation. In this context, agents such as heparins and therapies based on the protein C pathway, including activated protein C (APC), have been frequently explored for their potential to counteract the prothrombotic effects of NETs [209]. Because ROS can be rate-limiting for many NETosis programs, antioxidant strategies (e.g., N-acetylcysteine; NAC) or NADPH oxidase-targeting approaches (e.g., diphenyleneiodonium; DPI) represent an additional mechanistic category, aimed at preventing the oxidative burst that enables chromatin decondensation and granular enzyme trafficking [96,210].

Finally, a more unconventional concept is to suppress cancer-promoting NET activity using bacteriotherapy principles. Group A Streptococcus (GAS) has been discussed as a potential anti-cancer agent in PDAC, partly through its capacity to limit NET activity [211,212]. A proposed mechanism involves streptococcal collagen-like 1 (Scl1), which can reduce MPO activity and thereby interfere with a critical enzymatic step required for NET extrusion [213].

Current NET-targeting and NET-modulating therapeutic strategies in acute pancreatitis and PDAC, together with their mechanistic rationale and translational implications, are summarized in Table 3.

## 6. Future Research Directions and Knowledge Gaps

Despite significant progress in understanding NET biology, a fundamental limitation remains: there is still no standardized and clinically applicable method to measure NET burden or activity across different patient groups, timepoints, and research settings. Existing tools, such as measurements of cell-free DNA, citrullinated histone H3, MPO–DNA complexes, and co-localization imaging, provide valuable insights but are vulnerable to technical variability, sample handling issues, and biological confounders. For example, circulating DNA may originate from non-neutrophil sources, and the composition of NETs may vary depending on the trigger. Future research should focus on harmonizing protocols, validating composite NET scores across platforms, and developing longitudinal sampling approaches that can connect NET dynamics to outcomes in acute pancreatitis and to prognosis or treatment response in pancreatic cancer. This would align well with the growing interest in NET-based biomarkers while addressing the current limitations in assay standardization.

Another major gap involves the timing and causality of NET activity. Although NETs are strongly associated with disease severity and complications in acute pancreatitis, it remains unclear whether they are simply biomarkers or active contributors to pathology, and when exactly they shift from being potentially protective to harmful. The priming factors discussed in this review, including damage-associated molecular patterns, chemokines, interactions with platelets, oxidative and protease activity, and clearance mechanisms, likely act at different phases of the disease and in distinct clinical subtypes. This suggests that a uniform strategy for NET inhibition may not be effective in all cases. Future studies should incorporate time-resolved and compartment-specific analyses to distinguish NET formation in blood, pancreatic tissue, and distant organs. They should also explore different modes of NET release, such as lytic, vital, or mitochondrial, and investigate how NETs are cleared, whether by nucleases, macrophages, or other mechanisms. Clarifying these processes would help determine whether targeting key molecular mediators like PAD4, gasdermin D, MPO, neutrophil elastase, or autophagy offers greater therapeutic benefit than simply degrading extracellular DNA, especially in clinical settings where infection risk must be carefully managed.

In pancreatic cancer, the most pressing need is to better understand the spatial and functional context of NET formation. It is essential to determine where NETs are formed, whether in blood vessels, the stroma, or within tumor tissue, which cells trigger their release—tumor cells, fibroblasts, platelets, or endothelial cells—and which components of NETs contribute most to immune evasion, fibrosis, thrombosis, and metastatic progression. The concept of therapy-induced NET formation adds complexity, as treatment itself can alter cytokine signaling and neutrophil behavior. Therefore, NET biology in pancreatic cancer should be studied in relation to treatment timelines such as neoadjuvant, perioperative, and adjuvant phases. Advanced multi-omics tools, including spatial transcriptomics, single-cell and single-nucleus profiling, and high-dimensional imaging, combined with human-relevant models like organoids with immune and stromal co-cultures, microfluidic systems for thrombosis studies, and humanized mouse models, will be important for identifying which NET-related pathways are most therapeutically relevant.

On the therapeutic front, it is no longer sufficient to demonstrate that NETs are modifiable; the field must now show that targeting them yields selective clinical benefit. While the current literature outlines potential strategies such as degrading NETs with DNase, inhibiting upstream regulators like PAD4, oxidative pathways, or autophagy, and combining these with anticoagulant or immunotherapy approaches, the next step is to determine which patients are most likely to benefit. Future trials should be guided by biomarkers and focused clinical questions. For example, which patients have a NET-dominant inflammatory or thrombotic profile? Can early NET inhibition reduce organ failure in high-risk pancreatitis, or does it mainly prevent thrombo-inflammatory complications? In pancreatic cancer, could targeting NETs reduce perioperative venous thrombosis, improve immune infiltration into the tumor, and enhance the efficacy of checkpoint inhibitors or therapies directed at IL-8 and CXCR2, all without compromising antimicrobial defense or wound healing? These questions will require clinical studies with well-defined safety endpoints, infection monitoring, and pharmacodynamic evidence that the targeted pathways are being effectively modulated.

Beyond acute inflammation and cancer, a particularly underexplored but clinically meaningful next step is to extend NET biology into other exocrine pancreatic disorders, especially chronic pancreatitis, where neutrophil-driven programs may operate on longer, relapsing timescales. The key unanswered question is whether persistent or episodic NET formation is merely a byproduct of inflammatory activity or a causal driver of progressive parenchymal damage, ductal remodeling and stricturing, fibrogenesis, pain sensitization, and the gradual development of endocrine and exocrine failure, potentially through sustained crosstalk with macrophage-centered repair and pro-fibrotic pathways. Addressing this requires well-phenotyped chronic pancreatitis cohorts stratified by etiology, imaging-defined fibrosis burden, pain trajectories, and functional measures of exocrine reserve, coupled to standardized NET biomarker panels and, when feasible, tissue-level NET mapping in surgical specimens. In parallel, comparative mechanistic studies spanning acute pancreatitis, chronic pancreatitis, and PDAC could clarify which NET programs are shared versus disease-specific, and help define where NET-directed interventions might be most rational in practice, whether as anti-inflammatory modulation during recurrent flares, as an adjunct to limit fibrotic progression in established disease, or as a targeted strategy to mitigate vascular and thromboinflammatory complications in selected high-risk subgroups.

Finally, a conceptual gap persists. NETs are not a uniform entity but a family of extracellular chromatin–protein structures with diverse composition and context-dependent effects. Their protein content, formation pathways, and biological roles vary depending on the stimulus and disease setting. This supports the need for a more nuanced clinical classification of NETs, similar to the endotype-based approaches used in asthma or sepsis. Building such a framework in pancreatic diseases will require integration of NET markers with broader features of host response, such as platelet activity, coagulation profile, endothelial dysfunction, cytokine patterns, and signals from the gut microbiota. Testing whether these composite NET-related endotypes can predict clinical outcomes and guide treatment decisions would bring the field closer to the translational goal outlined in this review—aligning mechanism-based biomarkers with personalized, NET-targeted therapies.

## 7. Conclusions

Neutrophil extracellular traps represent a central mechanistic link in pancreatic disease, connecting local tissue injury with systemic complications in AP, and driving tumor-promoting inflammation, stromal remodeling, immune evasion, metastasis, and thrombosis in PDAC. The management of AP continues to rely on early, evidence-based supportive care, including goal-directed intravenous fluid resuscitation, adequate analgesia, and early enteral nutrition when feasible, alongside etiology-specific measures such as endoscopic retrograde cholangiopancreatography when biliary obstruction or cholangitis is present and timely cholecystectomy in gallstone-related disease, with antibiotics reserved for confirmed or suspected infected necrosis and minimally invasive step-up approaches for necrotizing complications. In PDAC, current standards remain stage-adapted and multimodal, encompassing surgical resection in resectable disease, perioperative or palliative systemic chemotherapy, selective radiotherapy, and biomarker-guided targeted therapies in defined subgroups. Within these frameworks, NET biology introduces a complementary mechanistic dimension: excessive NET formation can intensify microvascular dysfunction, thromboinflammation, and systemic complications in severe AP, while in PDAC it may reinforce stromal activation, immune exclusion, metastatic potential, and cancer-associated hypercoagulability, processes that are only partially addressed by existing therapies. Accordingly, strategies aimed at dismantling extracellular DNA scaffolds, inhibiting upstream regulators of NETosis such as peptidylarginine deiminase 4 or autophagy-related pathways, or mitigating downstream thromboinflammatory effects are best conceptualized as adjunctive interventions intended to dampen pathogenic amplification loops. Their clinical utility will likely depend on careful patient selection in NET-enriched contexts, such as AP with high risk of necrosis and organ failure or PDAC with prominent thromboinflammatory signatures, guided by harmonized biomarker panels including cell-free DNA, myeloperoxidase–DNA complexes, and citrullinated histone H3, and implemented with close attention to timing, safety, and rational combination with anticoagulant or immunomodulatory strategies.

## Figures and Tables

**Table 1:** NETs in acute pancreatitis: key mechanisms, biomarkers, and translational implications.

Topic	Key NET-Related Findings in AP	More Quantitative Evidence and Model Details	Representative Markers/Pathways	Clinical Significance/Therapeutic Implication	Key Take-home Message	References
AP severity biology	Dysregulated innate immune activation drives escalation from local acinar injury to SAP	Human epidemiology: ~34/100,000/year; SAP ~20–30% of cases; reported mortality in high-risk SAP cohorts 20–40%. Triggers: gallstones most common, then alcohol.	Neutrophil and macrophage recruitment, ROS, NE, MMP-9; NET components (DNA, histones, HMGB1)	Supports immune-targeted risk stratification and early recognition of patients at risk for SIRS, organ failure, and infected necrosis	SAP risk is largely immune-driven; neutrophils and NET programs sit on a critical escalation axis	[72,73,74,75,76,77,78,79,80]
Circulating NET biomarkers in humans	Circulating NET burden rises early and increases stepwise with severity; higher NET signatures associate with septic complications and worse outcomes	Clinical cohorts: AP vs. controls show higher cfDNA, DNA–histone, CitH3, MPO–DNA; CitH3 enriched in septic AP vs. non-septic, higher in ICU/fatal cases; kinetics: early rise post-admission.	cfDNA, DNA–histone complexes, CitH3, MPO–DNA	Candidate tools for early severity prediction, ICU triage, and sepsis-risk enrichment; potential composite panels with clinical scores	Blood NET signatures track severity and identify higher-risk AP trajectories	[87,94,95,96]
Tissue NETosis in experimental AP	NET deposition occurs in injured pancreas with systemic spillover; NET-rich deposits associate with extra-pancreatic inflammation (e.g., lung involvement)	Model: sodium taurocholate ANP. Histologic co-localization of extracellular DNA with elastase and histone markers (e.g., H2B). Systemic spillover: increased circulating cfDNA.	Pancreatic extracellular DNA with elastase and histone markers; increased systemic cfDNA	Mechanistic support for NETs as mediators of local injury and distant organ complications, not only bystanders	Experimental ANP shows true intrapancreatic NET structures with systemic dissemination	[87]
Recruitment–NET amplification loops	NETs promote neutrophil influx and activation via chemokine and adhesion programs; inhibiting NET generation lowers systemic inflammatory mediators	Model: experimental ANP. When NET generation inhibited: reduced circulating IL-6, HMGB1, CXCL2, MMP-9; mechanistic axes: increased pancreatic CXCL2 and increased Mac-1 on neutrophils with NET exposure; NET exposure increases ROS in isolated neutrophils.	CXCL2 induction; Mac-1 upregulation; IL-6, HMGB1, MMP-9 reduction when NETs inhibited	Defines actionable feed-forward circuits that could be targeted to blunt escalation from local inflammation to systemic injury	NETs are amplifiers: they recruit and prime more neutrophils, escalating inflammation	[87,97]
Upstream priming signals	Vascular activation promotes NETosis through adhesion-linked intracellular signaling that converges on PAD4-dependent chromatin remodeling	Human AP: P-selectin increased at presentation and correlates with clinical course. Mechanistic model: P-selectin binding PSGL-1 activates Syk and mobilizes Ca^2+^ (ER to cytosol), enabling PAD4 activity and histone citrullination. Experimental and human AP.	P-selectin–PSGL-1–Syk–Ca^2+^–PAD4 axis	Highlights anti-adhesion or upstream signal blockade as strategies to reduce NET formation and inflammatory propagation early in AP	Adhesion biology is not just trafficking; it biochemically licenses NET release via Ca^2+^–PAD4	[100,101]
Execution and DAMP amplification	NET release requires membrane permeabilization machinery; DAMPs can amplify NETosis and acinar activation loops	GSDMD increased in neutrophils in models and patients; inhibiting GSDMD reduces NETs and attenuates pancreatic injury/systemic inflammation/organ dysfunction in mice. eCIRP blockade reduces pancreatic CitH3/NET deposition and circulating DNA–histone complexes and lowers IL-6/HMGB1/MMP-9; eCIRP correlates with severity clinically.	GSDMD; eCIRP bound to NETs; downstream IL-6, HMGB1, MMP-9	Positions GSDMD and eCIRP as translational nodes for dampening NET output and systemic inflammation; potential biomarker plus target pairing	NETosis depends on execution machinery and is boosted by DAMP loops that sustain systemic inflammation	[102,103,104]
Resolution constraints	Pro-resolving lipid mediators can reduce neutrophil infiltration and PAD4-dependent NET markers, limiting disease severity	Model: PD1 (DHA-derived) reduces pancreatitis severity in mice; lowers neutrophil infiltration and CitH3; linked to lower PAD4 expression and reduced cfDNA/CitH3 release in vitro	Protectin D1 (PD1); reduced PAD4 expression; lower cfDNA/CitH3	Supports pro-resolving approaches as adjuncts to shift from injurious NET excess toward controlled resolution	Enhancing resolution can downshift PAD4-dependent NETosis and disease intensity	[105]
NET-driven acinar injury and ductal effects	NET histones promote trypsin activation and acinar stress signaling; NET aggregates can occlude ducts yet may also compartmentalize necrosis	Taurocholate models: NET interference reduces circulating MMP-9; NET exposure increases trypsin activity with STAT3 phosphorylation; histones (H2A/H2B/H3/H4; H4 prominent) drive effects via TLR9, Ca^2+^ oscillations, and STAT3. Ductal mechanism: PAD4-dependent intraductal NET aggregates are DNase-sensitive, enriched with serine proteases; PAD4 disruption reduces intraductal NETs and protects. Necrosis: aggregated NET layers may form barrier limiting DAMP diffusion and interfacing with coagulation and later fibrosis.	Histone H2A/H2B/H3/H4 (H4 prominent); TLR9, Ca^2+^ oscillations, STAT3; intraductal PAD4-dependent NET aggregates	Links NET products to early trypsin activation and ongoing injury; suggests dual role where reducing excessive aggregates may relieve obstruction while preserving needed containment	NETs can be direct effectors of pancreatic injury (biochemical and architectural), not only inflammatory byproducts	[87,106,107,108,109,110,111,112]
HTGP phenotype	HTGP associates with higher NET formation and worse clinical trajectory; gut microbiota metabolites modulate NET output via IL-17A skewing		PAD4; IL-17A; taurine; MAPK and NADPH oxidase modulation	Suggests microbiota-metabolite and IL-17A axes as targets for phenotype-specific risk reduction; supports exploring taurine-related or Th17-modulating strategies in HTGP	HTGP is a NET-prone, metabolically tuned phenotype where microbiota signals can tip IL-17A–NET amplification	[65,113,114,115]

**Table 2:** NETs in pancreatic malignancies (PDAC and pNETs): core mechanisms and clinical/therapeutic implications.

Core Topic	Key Take-Home Message (NETs in PDAC/Pancreatic Malignancies)	Key Markers/Nodes	Clinical Significance/Therapeutic Implication	Quantitative Evidence and Model Details	References
Disease context and innate dominance	PDAC is typically immune-cold and stroma-rich; neutrophils and NETs contribute to inflammatory yet immunosuppressive conditions that support progression.	NETs; neutrophil-rich TME; PSCs/CAFs	Supports stratifying “NET-high” tumors and considering NET biology as a functional driver of immune resistance and stromal remodeling.	Global burden context (GLOBOCAN 2022) and poor long-term outcomes provide the clinical backdrop for NET-focused mechanisms; immune-cold phenotype described with limited checkpoint benefit.	[116,117,118,119,120,121,122,123,124,125,126,127,128,129,130,131,132]
Experimental evidence of NET amplification	In vivo models show increased NETs in tumor and systemic compartments; tumor cells, fibroblasts, and platelets can all promote NET formation, sustaining feed-forward activation.	CAFs; platelets; tumor-derived cues; ROS-independent NET programs	Suggests multi-compartment targeting (tumor and circulation) and combination strategies that disrupt stromal and platelet–neutrophil cooperation.	Orthotopic murine PDAC models report increased NET abundance in tumor tissue and systemic compartments; rapid NET induction by tumor cells reported even when classical ROS dependence is not dominant; CAFs and platelets identified as NET-inducing components in the TME.	[120,133,134,135,136]
Clinical relevance and prognostic value	Patients often show elevated circulating NET markers, impaired clearance, and enriched intratumoral NETs; higher NET burden associates with worse survival and recurrence risk.	cfDNA; CitH3; MPO–DNA; tissue NETs	NET markers may support prognosis and recurrence-risk stratification, including alongside TNM, and may inform perioperative monitoring.	Human studies report increased circulating NET markers and impaired NET clearance; tumor sections show higher NET abundance vs. adjacent non-tumor; a large surgical series of resected PDAC linked tumor NET levels with worse postsurgical OS/RFS and improved risk stratification when combined with TNM. Post-pancreatectomy NET markers (cfDNA, CitH3) rise and peak several days postoperatively.	[120,133,137,138,139,140,141,142,143]
Tumor-intrinsic NET drivers	Tumor genetics and secreted factors can shape neutrophil recruitment and NETosis, including KDM6A-associated chemokine programs and TIMP1-linked signaling in neutrophils.	KDM6A; CXCL1/CXCR2; TIMP1; CD63; MEK/ERK	Highlights actionable axes (chemokine receptor targeting; TIMP1-associated pathways) and supports biomarker panels combining NET readouts with established markers.	KDM6A frequently inactivated in PDAC; KDM6A-deficient tumors upregulate CXCL1 (CXCR2 ligand) with associated TAN accumulation and higher NET formation; CXCL1 neutralization reduced neutrophil chemotaxis and NET-promoting activity in preclinical models. TIMP1 is elevated in PDAC tissue and spatially associated with NET-rich areas; proposed TIMP1–CD63–MEK/ERK axis supported by experimental disruption improving outcomes in models; circulating TIMP1 correlated with NET biomarkers in reported datasets	[11,144,145,146,147,148,149]
Stromal remodeling, invasion, and EMT	NET exposure promotes invasion and EMT-related signaling and can activate stellate cells, reinforcing desmoplasia and metastatic competence.	IL-1β; EGFR/ERK; HMGB1; RAGE; PAD4; IL-17	Provides rationale to evaluate NET targeting to reduce invasive behavior, stromal activation, and immune exclusion in NET-enriched disease.	In vitro NET-rich conditions enhanced migration/invasion; NET-associated IL-1β signaling linked to EGFR/ERK activation and EMT-related phenotypes; NET–stroma interaction includes PSC activation and desmoplastic reinforcement, with implications for liver micrometastatic seeding described in preclinical work.	[138,139,150,151,152,153,154]
Metastasis, thrombosis, and therapy resistance	NETs can facilitate metastatic seeding and contribute to cancer-associated thrombosis; chemotherapy can amplify neutrophil dominance and NET formation, promoting resistance programs.	CCDC25/ILK; CXCL8 (IL-8); STING–NF-κB; PAD4; TF; platelet activation	Supports integrated approaches combining NET modulation with anti-thrombotic and anti-inflammatory strategies and explores NET-blocking combinations to improve chemotherapy efficacy.	Metastasis: NETs capture circulating tumor cells and facilitate extravasation; pre-existing NETs in liver observed before detectable metastases in mouse models. Thrombosis: NETs act as coagulation scaffolds; xenograft and orthotopic models show reduced venous thrombosis with DNase I, neutrophil depletion, or PAD4 deletion. Therapy resistance: gemcitabine and GnP increase neutrophil infiltration and NET formation in mouse models; IL-8 surges after chemotherapy link NF-κB/STAT3 activation in tumor cells to CXCR1/2-driven NETosis; GPRC5A upregulation associated with IL-8 amplification and NLRP3-linked NET induction.	[150,151,152,167,168,180,181,182,183,184,185,186,187,188,189,194,195,196,197,198,199,200,201]

**Table 3:** NET-targeting and NET-modulating therapeutic strategies in acute pancreatitis and PDAC: mechanisms, evidence, and translational implications.

Strategy/Agent Class	Primary Target or Pathway	Proposed Anti-NET Mechanism	Context & Key Evidence (Summary)	Clinical Significance/Therapeutic Implication	Target Disease	Clinical Stage/Level of Evidence	References
DNase I	Extracellular DNA scaffold (NET backbone)	Degrades NET DNA backbone and destabilizes NET structure	In experimental SAP, NET dismantling associated with reduced pancreatic edema, lower histone deposition, and improved severity markers.	Rapid “downstream” neutralization of NET toxicity may reduce microvascular obstruction and protease/histone-mediated tissue injury; conceptually suited for acute, NET-rich inflammatory phases.	AP (especially SAP)	Preclinical (animal models)	[87]
PAD4 inhibition	PAD4/histone citrullination (CitH3)	Prevents chromatin decondensation required for NET extrusion	PAD4-dependent NET formation is a key amplification node; targeting PAD4 suppresses NET release in experimental settings	Mechanism-based prevention of NETosis; may reduce pancreatic necrosis progression and systemic inflammation	AP; PDAC	Preclinical (mechanistic/animal)	[202]
MPO–NE axis inhibition	MPO, NE (granule enzymes)	Blocks enzyme cooperation needed for chromatin remodeling and NET formation	Suppression of NET release and reduction in inflammatory injury markers in experimental systems	NET prevention approach; may attenuate local injury and cytokine escalation	AP	Preclinical (animal/in vivo)	[198,203]
Chloroquine (CQ)	Autophagy support for NETosis (incl. RAGE–autophagy axis)	Inhibits autophagic flux permissive for NET formation; reduces NET biomarkers	In SAP models: lower cfDNA/CitH3, reduced pancreatic injury, reduced IL-6 and HMGB1; in PDAC context: reduced NET markers and procoagulant/platelet activation signals	Repurposing candidate; mechanistically attractive where autophagy supports NETosis; potential to blunt thrombo-inflammation and perioperative VTE risk in PDAC	AP; PDAC	Preclinical + translational (animal models with patient-derived associations)	[96,120,203,204]
EGCG	NE activity and MPO-dependent chromatin remodeling; oxidative amplification	Suppresses NET formation; dampens oxidative/protease-dependent NET programs	Reported to suppress NET formation in vivo with less tissue injury and milder systemic inflammatory response.	Illustrates feasibility of pharmacologic NET suppression via protease/oxidant-linked pathways; potential adjunct concept to dampen inflammatory circuits in AP.	AP	Preclinical (animal models)	[205]
Thrombomodulin (TM)	HMGB1 neutralization/degradation; anticoagulant and anti-DAMP activity	Reduces NET-associated HMGB1 signaling implicated in EMT/inflammation and metastasis	In metastasis-focused PDAC model, proposed to reduce NET-driven dissemination by limiting extracellular HMGB1 signaling.	Translationally attractive where NET-associated DAMPs (HMGB1) support EMT and metastasis; may couple anti-inflammatory and antithrombotic benefits.	PDAC	Preclinical (disease models)	[151,172,207]
Antiplatelet strategies (e.g., ASA)	Platelet activation and platelet–neutrophil cooperation	Indirectly suppresses platelet–neutrophil cooperation required for robust NET induction	Proposed to indirectly suppress NET induction by reducing platelet-driven neutrophil priming in thrombo-inflammatory settings.	Potential to attenuate NET-associated immunothrombosis and vascular complications; conceptually relevant for PDAC hypercoagulability.	PDAC (thrombo-inflammatory context); potentially AP subsets	Mechanistic rationale/indirect evidence (largely non-NET interventional; NET-modulating inference)	[208]
Anticoagulants and protein C pathway (heparins, APC)	Thrombin and downstream coagulation propagation on NET scaffolds	Reduce thrombosis propagation on NET scaffolds; may limit NET-supported clot maturation	Considered to mitigate NET-driven thrombosis amplification by interrupting coagulation cascade support on NET structures.	Pragmatic approach to reduce VTE risk and clot stabilization in NET-rich PDAC; may complement NET-lowering strategies when thrombosis dominates.	PDAC (VTE risk); selected AP settings	Clinical use exists (anticoagulation), but NET-specific targeting: mixed/indirect	[209]
Redox-targeting (NAC, NADPH oxidase inhibition such as DPI)	ROS-dependent NETosis execution node	Prevent oxidative burst required for chromatin decondensation and enzyme trafficking	Proposed to limit oxidative burst required for chromatin decondensation and granular enzyme trafficking in many NET programs.	Mechanistic category aimed at preventing NET release across inflammatory contexts; supports combinatorial strategies where oxidative NETosis is dominant.	AP; PDAC	Preclinical (mechanistic); clinical readiness variable (agent-dependent)	[96,210]
Bacteriotherapy concept (GAS; Scl1)	MPO activity interference via bacterial factor (Scl1)	Reduces MPO activity and interferes with NET extrusion steps	Proposed anti-cancer approach partly through limiting NET activity by reducing MPO-dependent steps in NET extrusion.	Experimental, high-concept strategy that reframes NET suppression as an antitumor lever; currently best viewed as hypothesis-generating and preclinical.	PDAC	Preclinical/conceptual	[211,212,213]

## Data Availability

As this is a review article, no new data were generated or analyzed. All information supporting the conclusions of this work is derived from previously published studies duly cited in the manuscript. Therefore, a data availability statement is not applicable.

## Abbreviations

The following abbreviations are used in this manuscript:

2′3′-cGAMP	2′3′-cyclic guanosine monophosphate–adenosine monophosphate
3-MA	3-methyladenine
AJCC	American Joint Committee on Cancer
AKT	protein kinase B
ANCA	anti-neutrophil cytoplasmic antibody
ANP	acute necrotizing pancreatitis
AP	acute pancreatitis
APC	activated protein C
ASA	acetylsalicylic acid
ATP	adenosine triphosphate
Bcl-2	B-cell lymphoma 2
BPI	bactericidal/permeability-increasing protein
C5a	complement component 5a
CA19-9	carbohydrate antigen 19-9
CAFs	cancer-associated fibroblasts
Ca^2+^	calcium
CCDC25	coiled-coil domain-containing protein 25
CD11a/CD18	lymphocyte function–associated antigen 1 subunits
CD11b/CD18	Mac-1 subunits (β2-integrin)
CD177	cluster of differentiation 177
CD53	cluster of differentiation 53
CD54	intercellular adhesion molecule 1
CD62P	P-selectin
CD63	cluster of differentiation 63
CD8+	cluster of differentiation 8 positive
CitH3	citrullinated histone H3
COX	cyclooxygenase
CpG	cytosine–phosphate–guanine
CQ	chloroquine
CTCs	circulating tumor cells
CXCL1	C-X-C motif chemokine ligand 1
CXCL2	C-X-C motif chemokine ligand 2
CXCL5	C-X-C motif chemokine ligand 5
CXCL8	C-X-C motif chemokine ligand 8
CXCR1	C-X-C chemokine receptor 1
CXCR2	C-X-C chemokine receptor 2
DAMPs	danger-associated molecular patterns
DDR1	discoid domain receptor 1
DHA	docosahexaenoic acid
DNase	deoxyribonuclease
DNase I	deoxyribonuclease I
DNA	deoxyribonucleic acid
DPI	diphenyleneiodonium
ECM	extracellular matrix
ECs	endothelial cells
eCIRP	extracellular cold-inducible RNA-binding protein
EGCG	epigallocatechin-3-gallate
EGFR	epidermal growth factor receptor
EMT	epithelial–mesenchymal transition
ER	endoplasmic reticulum
ERK	extracellular signal-regulated kinase
ERK1/2	extracellular signal-regulated kinase 1/2
F-actin	filamentous actin
FIIa	thrombin (factor IIa)
FXa	factor Xa
GAS	group A streptococcus
G-CSF	granulocyte colony-stimulating factor
GLOBOCAN	Global Cancer Observatory cancer estimates
GnP	gemcitabine plus nab-paclitaxel
GPRC5A	G protein-coupled receptor class C group 5 member A
GSDMD	gasdermin D
GSK3β	glycogen synthase kinase 3 beta
H2A	histone H2A
H2B	histone H2B
H3	histone H3
H3K27me3	trimethylation of histone H3 lysine 27
H4	histone H4
HMGB1	high mobility group box 1
HTGP	hypertriglyceridemic pancreatitis
ICAM-1	intercellular adhesion molecule 1
IDO	indoleamine 2,3-dioxygenase
IF	immunofluorescence
IFN	interferon
IHC	immunohistochemistry
IL-1β	interleukin 1 beta
IL-6	interleukin 6
IL-8	interleukin 8
IL-17	interleukin 17
IL-17A	interleukin-17A
ILK	integrin-linked kinase
IPN	infected pancreatic necrosis
IRF3	interferon regulatory factor 3
ITGB1	integrin beta 1
KDM6A	lysine-specific demethylase 6A
LFA-1	lymphocyte function–associated antigen 1
LPS	lipopolysaccharide
Mac-1	macrophage-1 antigen (β2-integrin)
MAPK	mitogen-activated protein kinase
MEK	mitogen-activated protein kinase kinase (MAPK/ERK kinase)
MMP-9	matrix metalloproteinase 9
MPO	myeloperoxidase
mPTP	mitochondrial permeability transition pore
mtDNA	mitochondrial DNA
mTOR	mechanistic target of rapamycin
mTORC1	mechanistic target of rapamycin complex 1
mtROS	mitochondrial reactive oxygen species
NAC	N-acetylcysteine
NADPH	nicotinamide adenine dinucleotide phosphate
NE	neutrophil elastase
NET	neutrophil extracellular trap
NETosis	neutrophil extracellular trap formation
NETs	neutrophil extracellular traps
NF-κB	nuclear factor kappa B
NLR	neutrophil-to-lymphocyte ratio
NLRP3	NLR family pyrin domain containing 3
NK	natural killer
NOX2	NADPH oxidase 2
OPA1	optic atrophy 1
OS	overall survival
p-TBK1	phosphorylated TANK-binding kinase 1
PAF	platelet-activating factor
PaCa cells	pancreatic cancer cells
PAD4	peptidyl arginine deiminase 4
PANC-1	pancreatic cancer cell line
PARVB	beta-parvin
PD1	protectin D1
PD-L1	programmed death-ligand 1
PDAC	pancreatic ductal adenocarcinoma
PI3K	phosphoinositide 3-kinase
PKC	protein kinase C
PKCα	protein kinase C alpha
PKCθ	protein kinase C theta
PMA	phorbol 12-myristate 13-acetate
pNETs	pancreatic neuroendocrine tumors
PR3	proteinase 3
PS	phosphatidylserine
PSC	pancreatic stellate cell
PSCs	pancreatic stellate cells
PSGL-1	P-selectin glycoprotein ligand 1
RAF	rapidly accelerated fibrosarcoma kinase
RAGE	receptor for advanced glycation end products
RFS	recurrence-free survival
RNA	ribonucleic acid
ROS	reactive oxygen species
SAP	severe acute pancreatitis
Scl1	streptococcal collagen-like 1
SIRT1	sirtuin 1
SIRS	systemic inflammatory response syndrome
SLE	systemic lupus erythematosus
STAT3	signal transducer and activator of transcription 3
STING	stimulator of interferon genes
SYK	spleen tyrosine kinase
TAN	tumor-associated neutrophil
TANs	tumor-associated neutrophils
TBK1	TANK-binding kinase 1
TF	tissue factor
TGF-β	transforming growth factor beta
Th17	T helper 17
TIMP1	tissue inhibitor of metalloproteinases 1
TLR-2	Toll-like receptor 2
TLR-4	Toll-like receptor 4
TLR9	Toll-like receptor 9
TM	thrombomodulin
TNF-α	tumor necrosis factor alpha
TNM	tumor–node–metastasis
TME	tumor microenvironment
TSPAN25	tetraspanin 25
UTX	ubiquitously transcribed tetratricopeptide repeat X-linked
VTE	venous thromboembolism
ZEB1	zinc finger E-box-binding homeobox 1
α-SMA	alpha-smooth muscle actin
